# A standardized patient-derived glioblastoma organoid platform for evaluating radiotherapy efficacy and overcoming resistance

**DOI:** 10.1016/j.xcrm.2026.103095

**Published:** 2026-10-07

**Authors:** Xin Wang, Yusha Sun, Prithvi Sinha, Ruiying Su, Daniel Y. Zhang, Kristen H. Park, Yao Fu, Weifan Dong, Zhijian Zhang, Qing Wang, Daniel H. Geschwind, Riki Kawaguchi, Emily Ling-Lin Pai, Maria A. Gubbiotti, Robert Colbourn, Nduka M. Amankulor, Jay F. Dorsey, MacLean P. Nasrallah, Stephen J. Bagley, Zev A. Binder, Donald M. O’Rourke, Yi Fan, Guo-li Ming, Hongjun Song

**Affiliations:** 1 Department of Neuroscience and Mahoney Institute for Neurosciences, Perelman School of Medicine, University of Pennsylvania, Philadelphia, PA, USA; 2 Neuroscience Graduate Group, Perelman School of Medicine, University of Pennsylvania, Philadelphia, PA, USA; 3 Department of Radiation Oncology, University of Pennsylvania, Philadelphia, PA, USA; 4 Department of Neurosurgery, Perelman School of Medicine, University of Pennsylvania, Philadelphia, PA, USA; 5 Department of Biology, School of Arts and Sciences, University of Pennsylvania, Philadelphia, PA, USA; 6 Program in Neurogenetics, Department of Neurology, David Geffen School of Medicine, University of California, Los Angeles, Los Angeles, CA, USA; 7 Department of Pathology and Laboratory Medicine, Perelman School of Medicine, University of Pennsylvania, Philadelphia, PA, USA; 8 Glioblastoma Translational Center of Excellence, The Abramson Cancer Center, University of Pennsylvania, Philadelphia, PA, USA; 9 Division of Hematology and Oncology, Department of Medicine, Perelman School of Medicine, University of Pennsylvania, Philadelphia, PA, USA; 10 Center for Cellular Immunotherapies, Perelman School of Medicine, University of Pennsylvania, Philadelphia, PA, USA; 11 Parker Institute for Cancer Immunotherapy, Perelman School of Medicine, University of Pennsylvania, Philadelphia, PA, USA; 12 Institute for Regenerative Medicine, University of Pennsylvania, Philadelphia, PA, USA; 13 Department of Cell and Developmental Biology, Perelman School of Medicine, University of Pennsylvania, Philadelphia, PA, USA; 14 Department of Psychiatry, Perelman School of Medicine, University of Pennsylvania, Philadelphia, PA, USA; 15 The Epigenetics Institute, Perelman School of Medicine, University of Pennsylvania, Philadelphia, PA, USA

**Keywords:** glioblastoma, organoids, radiotherapy, transcriptomic signature, tumor heterogeneity, BET inhibitors, radiotherapy resistance, combinatorial therapy

## Abstract

Patient-derived glioblastoma (GBM) organoids (GBOs) maintain characteristics of their original tumors; however, they have not been widely applied to systematically assess therapy responses at scale or elucidate mechanisms underlying treatment resistance. Here, we develop a standardized reaggregated GBO (rGBO) platform for scalable, quantitative evaluation of radiation responses across 48 patients with GBM. Radiation responses in rGBOs correlate with the survival duration of corresponding patients treated with standard-of-care radiotherapy. Transcriptomic analyses identify gene signatures associated with radiosensitivity and radioresistance. Connectivity mapping with perturbational datasets identifies a panel of compounds with potential for reducing radioresistant and enhancing radiosensitive signatures. Subsequent testing demonstrates that inhibitors of bromodomain and extraterminal (BET) family proteins enhance radiotherapy efficacy in GBO and xenograft models by downregulating the radioresistant signature. Together, our study highlights the potential of rGBOs for standardizing therapeutic screening, evaluating patient-specific responses, identifying associated molecular features, and developing strategies to overcome treatment resistance.

## Highlights

• Standardized rGBO platform for evaluating differential radiotherapy responses at scale
• Correlation between rGBO radiation responses and survival time of matched patients
• Transcriptomic signatures differentiating radiosensitive and radioresistant rGBOs
• Enhancement of radiation efficacy by BET inhibitors in GBO and xenograft models

## Graphical abstract

**Figure:**
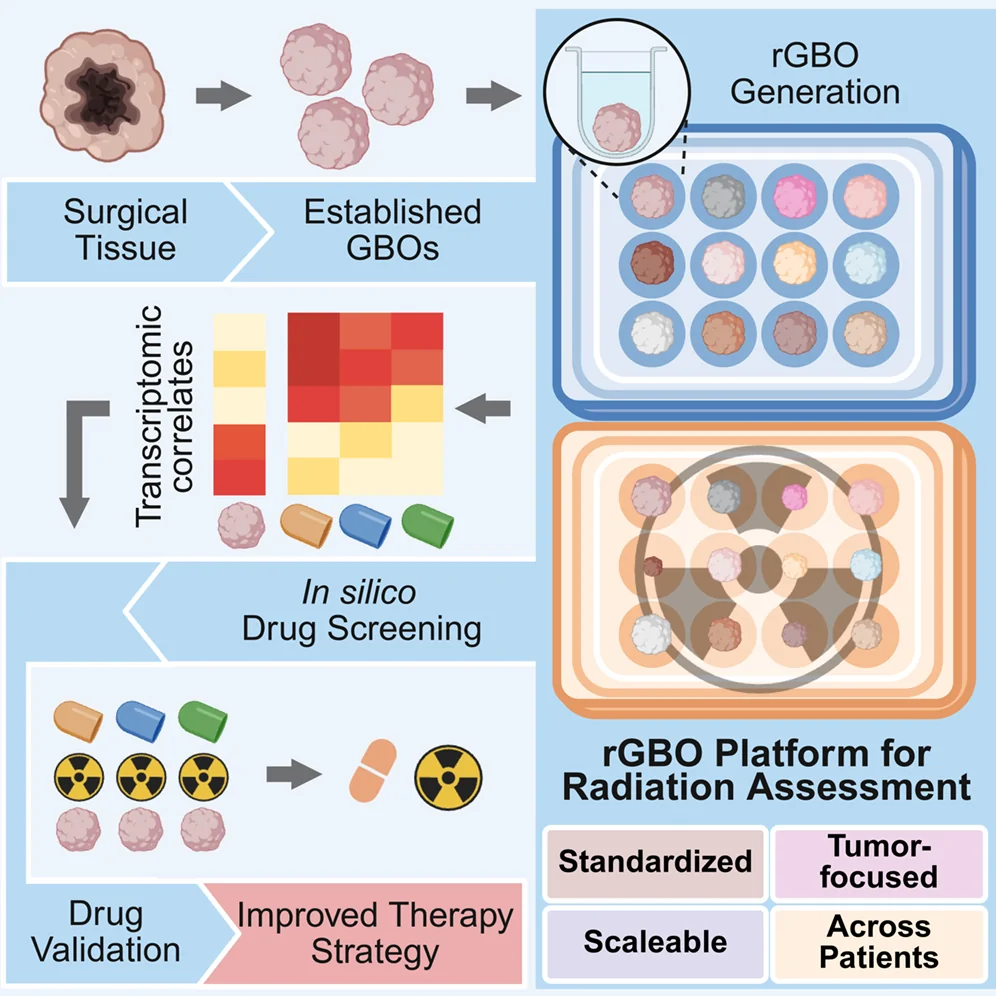

## Teaser

Wang et al. develop a standardized reaggregated glioblastoma organoid (rGBO) platform that enables scalable evaluation of patient-specific treatment responses. The platform links organoid radiosensitivity with patient survival, identifies response-related transcriptomic signatures, and reveals BET inhibition as a strategy to enhance radiotherapy efficacy in organoid and xenograft models.

## Introduction

Glioblastoma (GBM) is a highly aggressive brain tumor in adults characterized by significant inter- and intratumoral heterogeneity,^1^^,^^2^^,^^3^ which contributes to treatment resistance and recurrence.^4^^,^^5^^,^^6^ Standard-of-care therapy consists of maximal surgical resection followed by chemotherapy and radiotherapy,^7^ eliciting variable responses across patients.^8^ Beyond O^6^-methylguanine-DNA methyltransferase (*MGMT*) promoter methylation,^9^ the mechanisms underlying differential responses to standard-of-care radiotherapy remain elusive. Large-scale profiling studies have elucidated molecular changes in recurrent tumors,^5^^,^^10^ but these are often confounded by treatment effects. Studies proposing several resistance mechanisms to radiotherapy, such as upregulation of DNA damage repair^11^ or enrichment of glioma stem cells,^12^ were largely restricted to a limited number of cell lines and may not capture patient-level heterogeneity. These limitations highlight the necessity for high-fidelity preclinical models that recapitulate patient-specific responses to investigate mechanisms driving treatment resistance.

Patient-derived tumor organoids are increasingly recognized as essential tools for cancer research, providing a more accurate and physiologically relevant representation of patient tumors.^13^^,^^14^^,^^15^^,^^16^^,^^17^^,^^18^^,^^19^ Recent studies using tumor organoids for treatment modeling and personalized medicine across multiple cancers have shown promise in recapitulating clinical outcomes and advancing clinical guidance.^20^^,^^21^^,^^22^^,^^23^^,^^24^ Patient-derived GBM organoids (GBOs),^13^^,^^14^ explants,^25^ and co-culture systems^26^ offer distinct advantages over traditional monolayer or sphere cultures, including rapid establishment, growth factor- and serum-free conditions, and improved preservation of tumor heterogeneity and the tumor microenvironment (TME), making them powerful platforms for therapeutic screening and translational research.^27^^,^^28^^,^^29^^,^^30^ Distinct treatment responses have been observed between 3D and 2D monolayer cultures,^31^^,^^32^ yet very few studies using conventional 2D or sphere-based assays, or tumor organoids have modeled therapeutic efficacy to demonstrate correlations with patient clinical outcomes in GBM in a large sample size.^26^^,^^27^^,^^29^^,^^33^^,^^34^ For example, two studies using 22 and 18 patients respectively demonstrated that individualized GBM organoids recapitulate temozolomide (TMZ) responses consistent with *MGMT* promoter methylation and post-treatment patient survival.^26^^,^^35^ We recently employed GBOs alongside a phase 1 clinical trial of dual-targeting chimeric antigen receptor (CAR) T cells in patients with recurrent GBM.^36^ In that study of six patients, co-culture of GBOs with autologous patient CAR T products recapitulated multiple response metrics observed in matched trial patients, highlighting the potential of GBOs as avatars to prospectively model heterogeneous clinical treatment responses.^27^ While these studies show promise, they are limited by small cohort sizes and lack standardized generation procedures to allow scalability.

Here, we present a reaggregated GBO (rGBO) model, in which established GBOs without the TME are digested and reaggregated at defined cell numbers, yielding a standardized tumor cell aggregate model that resolves inter-organoid variability and enables scalable assessment of tumor responses to therapy across different patients. We comprehensively mapped radiotherapy responses of rGBOs from 48 patients and found a significant correlation between rGBO radiosensitivity and patient survival time following surgery. By integrating tumor transcriptomic profiles, we identified gene signatures associated with differential radiotherapy responses. As a proof-of-principle for the approach, we further identified and validated compounds capable of modulating these signatures to enhance radiotherapy efficacy. Overall, our study demonstrates the utility of a standardized rGBO model for quantitative and scalable assessment of heterogeneous radiotherapy responses across GBM patients, identifies molecular signatures associated with radiosensitivity and radioresistance, and provides a framework for discovering more effective therapeutic strategies.

## Results

### Standardized rGBOs for scalable quantitative testing

To reduce the variability of GBOs^1^^,^^13^ and to standardize the GBO model for scalable assays with quantitative measurements, we established a method to dissociate GBOs into single cells using a commercial brain tumor dissociation kit and reaggregated them with a defined cell number by using ultra-low attachment 96-well U-bottom plates (Figure S1A; 5,000 cells per well). GBOs cultured for more than 2 months with minimal TME components were used to yield a simplified model for focused assessment of tumor responses to therapy using luminescent ATP detection assay for high-throughput measurement of cell viability. During overnight static culture, cells in each well formed 3D tumor aggregates, referred to as rGBOs (Figure S1B). The average live cell percentage after dissociation was above 70% for all GBOs tested regardless of culture duration, indicating robust establishment of the rGBO model (Figure S1C).

We first assessed the ability of our rGBO approach to reduce inter-organoid variability by using GBOs from a patient carrying EGFRvIII, a well-established EGFR variant with heterogeneous expression in GBM.^37^^,^^38^ EGFRvIII staining revealed pronounced heterogeneity among different GBOs derived from the same patient, whereas matched rGBOs showed a markedly reduced variance in the proportion of EGFRvIII^+^ cells (Figures S1D and S1E).

To evaluate preservation of tumor cell subtypes in rGBOs, we compared subtype marker expression and transcriptomic profiles between rGBOs and matched GBOs. Immunohistochemistry showed comparable expression of canonical GBM subtype markers (Figures S1F and S1G), while RNA sequencing (RNA-seq) revealed highly concordant GBM subtype scores between rGBO and matched GBO samples (Figures S1H–S1J). Accordingly, single-cell RNA-seq of paired GBOs and rGBOs showed that rGBOs preserve major GBM cellular states (Figures S1K–S1N).^1^^,^^39^

Collectively, these results indicate that rGBOs closely resemble matched GBOs at both protein and transcriptomic levels while exhibiting reduced inter-organoid variability, supporting their use as a standardized model for scalable analyses.

### Differential radiotherapy responses of rGBOs

To evaluate the validity of rGBOs to model patient treatment responses, we turned to radiotherapy, which is a standard-of-care treatment following surgery for almost all patients with GBM. In total, we examined samples from 48 patients (Table S1; primary samples *n* = 32; recurrent samples, *n* = 16). We tested radiation efficacy on rGBOs from these patients and compared their efficacy based on patient time to death following surgery, if available. In addition, we performed RNA-seq on matched rGBOs, GBOs, and primary tissues in parallel to identify transcriptomic features associated with differential radiation responses in rGBOs. We extracted molecular signatures from the discovery cohort (22 patients) and validated these signatures in the second cohort (26 patients) (Figure 1A). We further explored these signatures to identify compounds that might modulate these signatures and enhance radiation efficacy in rGBOs and after xenotransplantation (Figure 1A).

**Figure 1:**
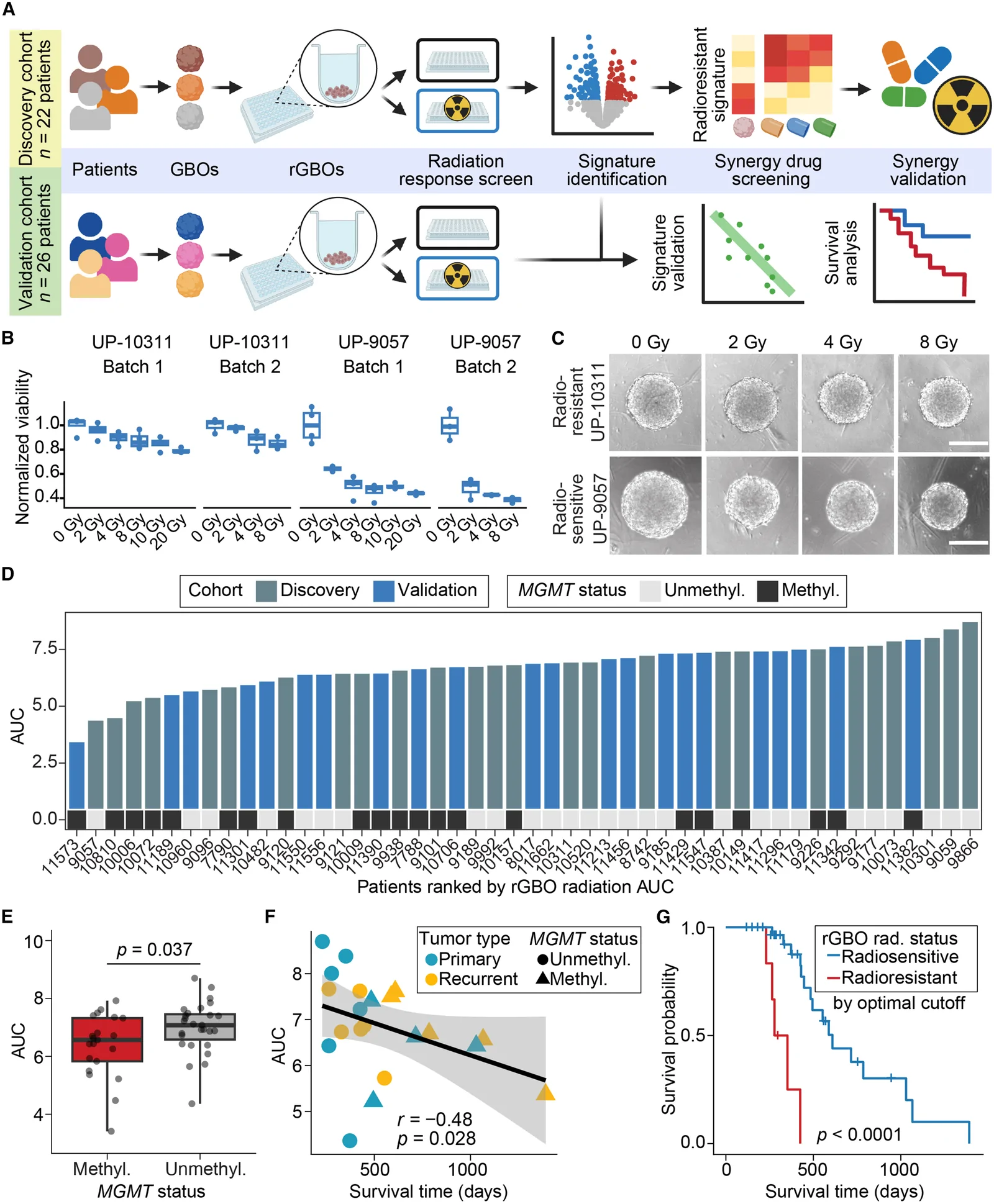
Differential radiotherapy responses in rGBOs (A) Illustration of the procedures to map radiotherapy responses in rGBOs followed by transcriptome analysis and identification of compounds for synergistic effects. (B) Quantitative analysis of rGBO cell viability at 4 days post-radiotherapy (2, 4, and 8 Gy, normalized to 0 Gy condition) from two batches with a culture time difference of over 3 months. (C) Sample bright-field images of rGBOs that were sensitive or resistant to radiotherapy at 4 days post-radiation at different doses. Scale bars, 200 μm. (D) Ranked area under the curve (AUC) values of viability response curves to radiotherapy in rGBOs derived from *n* = 48 patients. Patient *MGMT* promoter methylation is indicated underneath. Unmethyl., unmethylated; methyl., methylated. (E) Quantification of AUC values based on patient *MGMT* status with a two-tailed Wilcoxon test (unmethylated, *n* = 27 patients; methylated; *n* = 21 patients). (F) Spearman correlation analysis of patient survival time (time to death after surgery, days) with radiation responses of matched rGBOs, measured by the AUC values of the cell viability curves (*n* = 21 patients deceased before time of data collection and >100-day survival post-surgery). (G) Kaplan-Meier survival curve for GBM patients (*n* = 38 patients with >100-day survival post-surgery) stratified by radiotherapy sensitivity in rGBOs. The optimal cutoff is determined via maximally selected rank statistics. Statistical significance is assessed using log rank tests. See also Figures S1 and S2; Table S1.

We applied radiation doses of 2, 4, 8, 10, and 20 Gy to rGBOs with at least three replicates per condition. After 4 days, we measured cell viability by using a luminescent ATP detection assay, and the results were normalized to the control condition without radiation. We found that cell viability was highly concordant among replicates (Figure 1B). Moreover, cell viability decreased with increasing radiation doses, demonstrating the efficacy of radiotherapy (Figure 1B). We measured the radiotherapy response of rGBOs from the same patient at different culture durations and observed consistent results (Figure 1B, culture time gap >3 months between two batches), suggesting that the radiotherapy response of GBOs from the same patient remains intrinsically stable in culture over the time window we examined and across biological replicates. Since 10 and 20 Gy radiation did not result in significantly greater effects, we only used doses of 0, 2, 4, and 8 Gy in subsequent experiments (Figure 1B). As an orthogonal validation, rGBO volume, as shown by bright-field images, mirrored the cell viability patterns across different patients (Figure 1C). Together, these results suggest that rGBOs provide a reproducible high-throughput platform for quantitatively measuring radiotherapy responses via cell viability.

We next examined rGBOs from 48 patients with diverse genetic mutations (Table S1). We calculated an area under the curve (AUC) metric to integrate the responses across all radiation doses (a higher AUC indicates greater radioresistance). We ranked the radiation responses of rGBOs, which exhibited substantial variation among patients (Figures 1D and S2A). As radiation sensitivity in GBM correlates with *MGMT* promoter methylation,^40^ we integrated rGBO radiotherapy responses with matched patient *MGMT* promoter methylation status and observed significantly greater sensitivity when methylation was present (Figures 1D and 1E). To further validate the ability of rGBOs to model patient clinical outcomes, we correlated rGBO radiation responses with matched patient survival time after surgery. We found a significant association, with more sensitive rGBOs corresponding to longer patient survival (Figure 1F). Stratification of patients by rGBO radiation response also revealed a significant difference in patient survival time, as shown by Kaplan-Meier survival analyses using both optimal cutoff and top and bottom thirds of responses (Figure 1G; Figures S2B and S2C). We confirmed that rGBO responses were independently associated with patient survival time after adjusting for covariates including *MGMT* promoter methylation, recurrence status, and *TP53* mutation (Figure S2D). Notably, rGBO-defined radiosensitivity significantly stratified survival time within both primary and recurrent patient subgroups (Figure S2F). The correlation between rGBO radiotherapy response and patient survival time remained significant in the recurrent subgroup and showed a similar but non-significant trend in primary GBM, likely due to the smaller sample size (Figure S2E). Together, our results with a larger cohort showed that rGBOs effectively capture differential radiotherapy responses in GBM, with significant correlations to survival time of patients who received radiation therapy.

### Radiosensitive and radioresistant gene signatures

To investigate molecular features underlying differential radiotherapy responses, we performed RNA-seq on pre-radiation samples from GBOs, matched rGBOs, and primary tissues to capture intrinsic determinants of radiotherapy resistance and avoid confounding radiation-induced effects (Table S2A). We first examined the discovery cohort (*n* = 22 patients) and identified differentially expressed genes (DEGs) with a statistically significant linear trend with the radiation AUC variable in rGBOs using DESeq2,^41^ adjusting for factors such as batch effects and sample type. A total of 339 DEGs were negatively associated with radiation AUC (radiosensitive signature), while 753 DEGs were positively associated with radiation AUC (radioresistant signature) (Figure 2A; Tables S2B and S2C). Further examination of individual DEGs confirmed consistent patterns across sample types, reinforcing the transcriptomic similarity between different sample types and their reliability in identifying molecular signatures for radiation responses (Figures 2B and 2C). For example, the expression of Dickkopf WNT signaling pathway inhibitor 1 (*DKK1*) was found to be positively correlated with radiotherapy resistance of rGBOs (Figure 2B), consistent with previous findings that DKK1 can induce resistance to radiotherapy and immunotherapy in head and neck squamous cell carcinoma.^42^ In contrast, protocadherin gamma subfamily B 6 (*PCDHGB6*), which encodes a member of the protocadherin family involved in cell-cell adhesion and has been reported to exhibit methylation alterations associated with solid tumors,^43^ was found to be positively correlated with radiotherapy sensitivity (Figure 2C). Network analyses using Metascape^44^ showed that the radioresistant DEGs were enriched in terms related to inflammatory response/cytokine signaling as well as extracellular matrix (ECM) organization, both of which are known to be linked to radiotherapy resistance and GBM progression (Figure 2D).^45^^,^^46^ Notably, we also identified less-characterized terms and pathways related to radiotherapy resistance, such as nuclear receptors (Figure 2D). In addition, Gene Ontology (GO) analysis of radioresistant DEGs confirmed significant enrichment in terms related to immunoreactive processes, such as cytokine-mediated signaling pathway and regulation of inflammatory response (Figure 2E).

**Figure 2:**
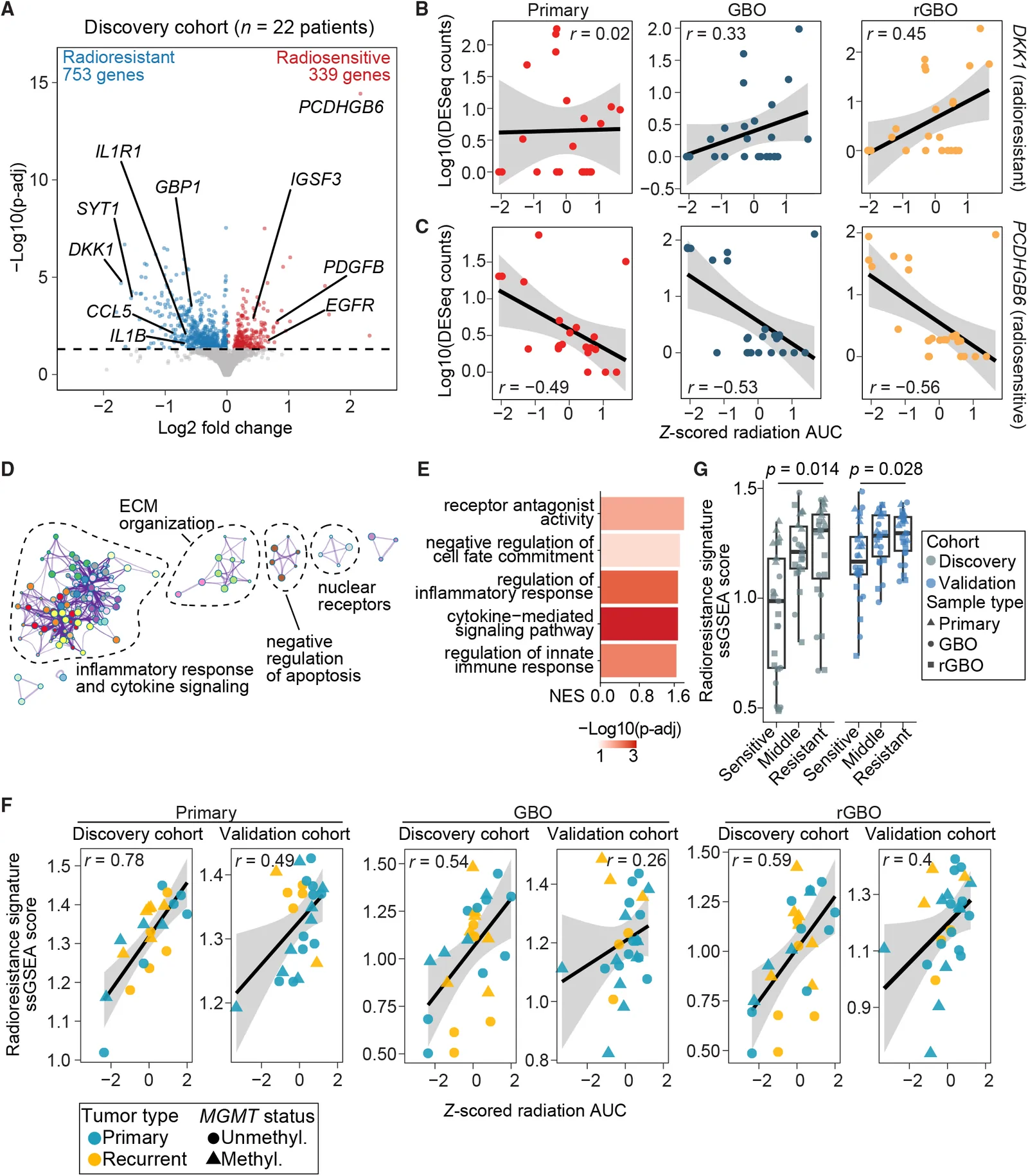
Transcriptomic signatures associated with differential radiotherapy responses in rGBOs (A) Volcano plot showing DEGs whose expression levels significantly correlate with radiotherapy responses in rGBOs. Red dots represent genes associated with radiotherapy sensitivity, while blue dots indicate genes linked to radiotherapy resistance. Log2 fold change values represent the change in gene expression per unit decrease in radiation AUC, controlling for sample type, *MGMT* promoter methylation, and tumor recurrence. (B and C) Correlation analyses between the expression of one example radioresistant DEG, *DKK1* (B), one example radiosensitive DEG, *PCDHGB6* (C), across different sample types (primary tissue, GBO, and rGBO) and the radiotherapy AUC values from matched rGBOs. (D and E) Network analysis (D) and enriched GO terms (E) of the radioresistant DEGs. Normalized enrichment score (NES) of GO terms is shown on the *x* axis. (F) Correlation analyses between single-sample gene set enrichment analysis^47^ (ssGSEA) scores of the identified radioresistant signatures across different sample types (primary tissue, GBO, and rGBO) and *Z*-scored AUC values of radiotherapy response from matched rGBOs from both discovery and validation cohorts. (G) Boxplots showing the expression of identified radioresistant signature, stratified by rGBO radiotherapy responses (sensitive: bottom 33.3%, middle: 33.3%–66.7%, resistant: top 33.3%). Data points are colored by the cohort, and point shapes indicate the sample type. In order to account for samples from the same patient but different sample types, we computed *p* values via a linear mixed-effects model fit by maximum likelihood, with patient number as a random variable. See also Figure S3; Table S2.

We next examined our validation cohort. For both discovery and validation cohorts, a larger response to radiotherapy tended to correspond to either decreased radioresistant or increased radiosensitive signatures (Figure 2F; Figure S3A). Additionally, we stratified patients into thirds based on their rGBO-profiled radiotherapy responses and compared their expression of radioresistant or radiosensitive gene signatures, which revealed significant differences between the top and bottom groups in both discovery and validation cohorts (Figure 2G; Figure S3B). Together, these analyses identified intrinsic transcriptomic signatures associated with radiosensitivity and radioresistance in tumor cells and demonstrate that their expression is consistently linked to rGBO-profiled radiotherapy responses across discovery and validation cohorts.

### Screening of compounds modulating radioresistant and radiosensitive signatures

Instead of targeting individual genes within the radioresistant and radiosensitive signatures to assess their impact on treatment efficacy in rGBOs, we explored the possibility to identify compounds that modulate the broader landscape of gene signatures associated with radiotherapy responses. To achieve this, we utilized perturbational datasets, including the CmapR package^48^ and the ConnectivityMap platform,^49^ which provide gene expression profiles affected by treatment of many compounds. We systematically searched for compounds capable of reversing the radioresistant signature and/or enhancing the radiosensitive signature (Figure 3A). This approach identified several compounds, ranked by their efficacy scores (Table S3A). We then prioritized compounds based on consistent molecular mechanisms of action (Figure 3A) and included additional compounds targeting concordant pathways with the potential ability to penetrate the blood-brain barrier (BBB) to facilitate *in vivo* analysis. Additionally, inhibitors targeting several individual DEGs, such as *DKK1*, C-X-C motif chemokine ligand 8 (*CXCL8*), and C-X-C motif chemokine receptor 2 (*CXCR2*), were included for comparison. Through this prioritization pipeline, we curated a list of 15 candidate compounds for functional validation (Table S3B). These compounds were tested at multiple concentrations for their ability to enhance radiation sensitivity in rGBOs from different patients. We calculated zero interaction potency (ZIP) synergy scores by using viability values collected at 0 and 8 Gy radiation doses (Figure 3B). From this analysis, bromodomain and extraterminal (BET) family protein inhibitors, including JQ-1, PFI-1, and I-BET151, demonstrated generally greater synergy with radiation in most rGBOs tested, with JQ-1 showing particularly strong effects (Figure 3B; Table S3C). Notably, XMD8, which also exhibited synergy with radiotherapy (Figure 3B), acts as a dual inhibitor of extracellular signal-regulated kinase 5 (*ERK5*) and the BET protein bromodomain-containing protein 4 (*BRD4*).^50^ In contrast, selected inhibitors targeting three individual DEGs demonstrated lower efficacy compared with those identified from the perturbational datasets (Figure 3B), suggesting that targeting broader gene signatures may be a more effective strategy for drug selection.

**Figure 3:**
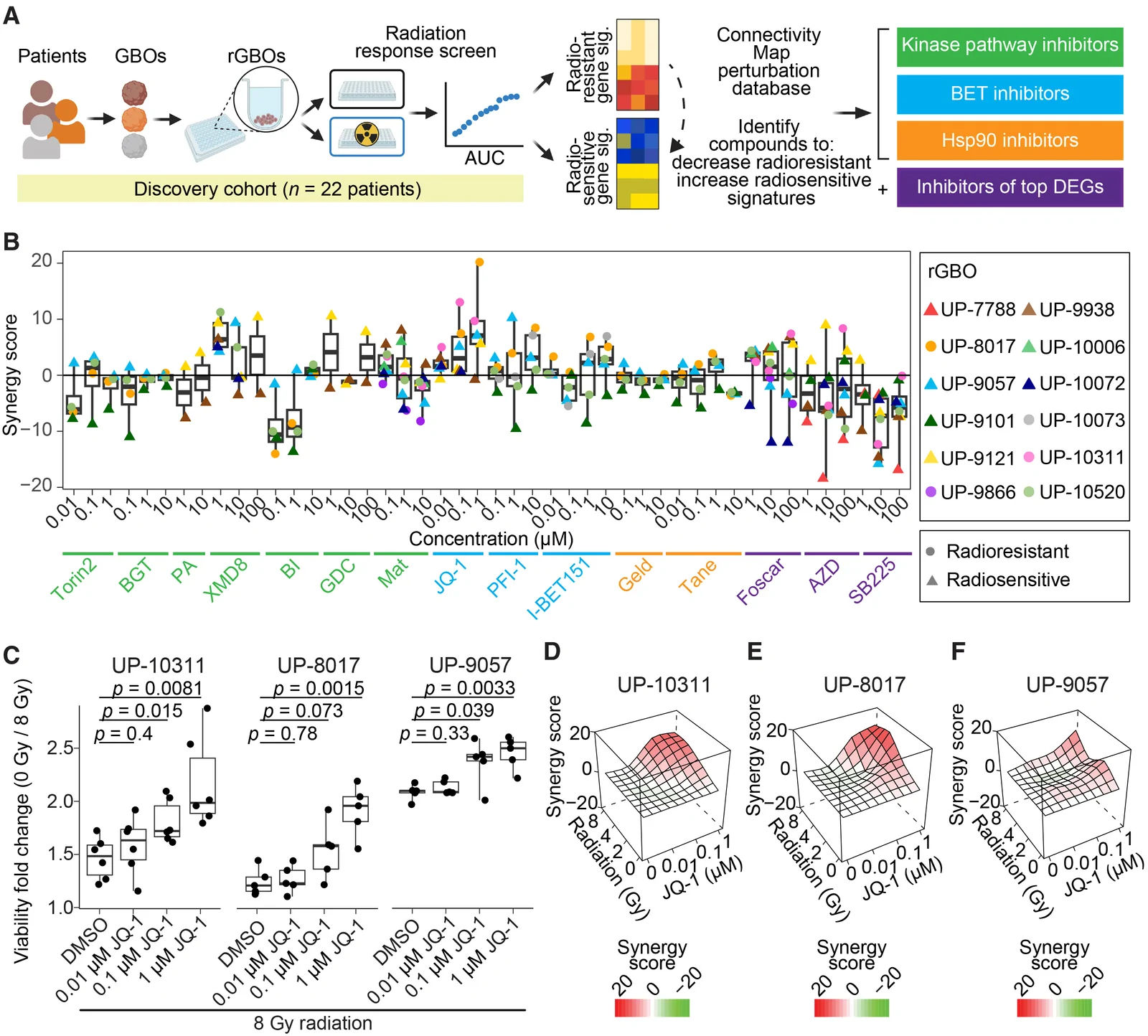
Enhancing radiotherapy efficacy in rGBOs with a BET inhibitor (A) Schematic illustration of *in silico* screening of synergistic compounds that modulate radioresistant or radiosensitive gene signatures, along with the molecular mechanisms of action of the resulting compounds (right). sig., signature. (B) Boxplots showing zero interaction potency (ZIP) synergy scores calculated using viability values at 0 and 8 Gy with candidate compounds at different concentrations, normalized to the values at 0 Gy with DMSO treatment. BGT, BGT226; PA, paxalisib; XMD8, XMD8-92; BI, BI-2563; GDC, ipatasertib; Mat, masitinib; Geld, geldanamycin; Tane, tanespimycin; Foscar, foscarnet; AZD, AZD-5069; and SB225, SB225002. Colors used for the compound names (text) and the bars below the *x* axis correspond to the compound categories depicted in (A). (C) Fold change in viability of rGBOs under 0 Gy (non-irradiated) conditions relative to corresponding 8 Gy-irradiated rGBOs, across varying concentrations of JQ-1. Each point represents an independent biological replicate derived from separate dissociation/reaggregation of rGBOs from three patients. Statistical significance is assessed using two-sided Welch’s *t* tests (*n* = 5). (D–F) 3D surface plots showing the ZIP synergy score of JQ-1 treatment and radiation of rGBOs (D, UP-10311; E, UP-8017; F, UP-9057). See also Figure S3; Table S3.

We further evaluated JQ-1 under varying radiation doses and observed consistent synergistic effects across rGBOs from multiple patients (Figures 3C–3F; Table S3D). To confirm the involvement of BET family proteins in radiotherapy synergy in rGBO responses, we examined two additional BET inhibitors,^51^ ABBV-744 and GSK778, and observed a consistent synergistic effect in rGBOs from different patients (Figures S3C–S3F; Table S3E). Additionally, we validated the synergy between JQ-1 and radiation by directly applying the treatments to whole GBOs without reaggregation, and we also observed synergy for GBOs from different patients (Figures S3G–S3J). Together, these results identify BET inhibitors as a potential combinatory treatment to enhance the efficacy of radiotherapy in GBM.

### Downregulation of the radioresistant signature by a BET inhibitor

We next investigated the mechanism underlying radiotherapy synergy observed with BET inhibitors. We performed RNA-seq on rGBO samples from 4 patients treated with JQ-1 at effective concentrations of 0.1 and 1 μM, along with a DMSO control. After adjusting for batch and patient effects, we found that JQ-1 treatment at both concentrations induced substantial transcriptomic changes across rGBOs, resulting in 4,979 downregulated and 5,123 upregulated DEGs at 1 μM, and 3,257 downregulated and 1,324 upregulated DEGs at 0.1 μM, compared with the DMSO control (adjusted *p* value <0.05 and |log_2_Foldchange| > 0) (Figure 4A; Tables S4A and S4B). Most of the DEGs exhibited the same directional changes by both concentrations of JQ-1, implying that the mechanisms and targets induced by JQ-1 are consistent across different concentrations (Figure 4A). Importantly, we confirmed a significant downregulation of the radioresistant signature with JQ-1 treatment (Figures 4B and 4C; Figures S4A and S4B), validating the utility of leveraging perturbational datasets to tune transcriptional landscapes. We also compared the expression of the radioresistant signature and several individual components with published BRD-regulated programs in GBM^52^^,^^53^ and showed consistent downregulation by JQ-1 (Figure S4C). GO analyses revealed that downregulated DEGs were enriched for ECM, immune regulation, and inflammation, which align with terms derived from the radioresistant signature, whereas upregulated DEGs were enriched for terms related to chromatin and nucleosome organization (Figures 4D and 4E; Figures S4D and S4E). We validated the downregulation of a representative ECM molecule, Tenascin C, by JQ-1 treatment in GBOs from three patients at the protein level (Figures S4F and S4G). Together, these findings suggest that BET inhibitor JQ-1 enhances radiotherapy efficacy by suppressing the radioresistant program, including ECM and other mesenchymal programs, thereby reprogramming GBM toward a more radiosensitive state.

**Figure 4:**
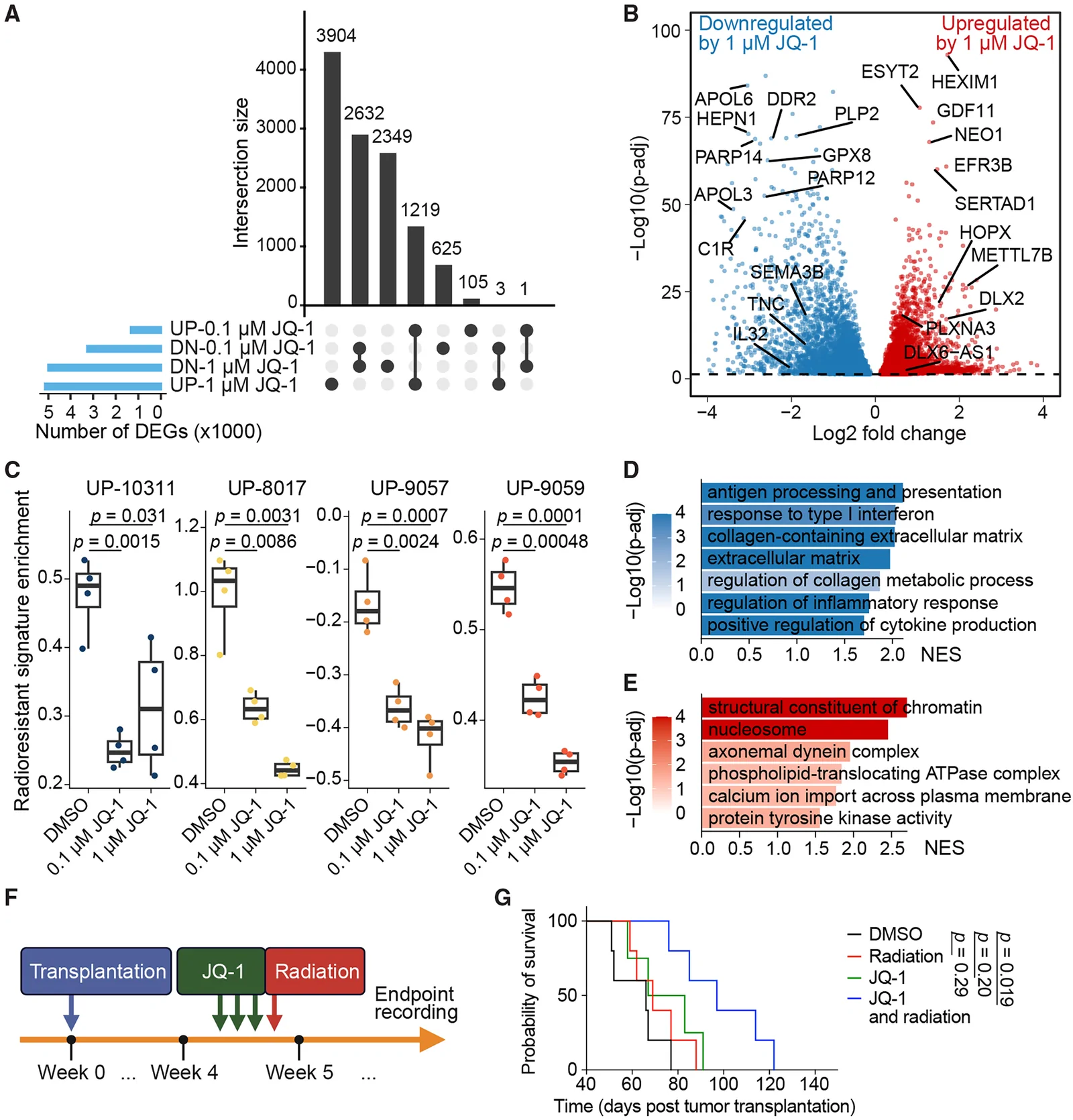
JQ-1 synergizes with radiotherapy via downregulation of the radioresistant signature (A) Upset plot showing the interaction size of different upregulated or downregulated DEGs from 0.1 μM JQ-1 or 1 μM JQ-1 treatment compared with DMSO control (rGBOs from *n* = 4 patients, 4 replicates per condition). (B) Volcano plot showing upregulated (red) or downregulated (blue) DEGs following 1 μM JQ-1 treatment compared with the control condition. (C) Boxplots showing signature scores grouped by different conditions for rGBOs from *n* = 4 patients. Statistical significance is assessed using two-tailed Student’s *t* test. (D and E) GO terms enriched for downregulated (D) and upregulated (E) DEGs following 1 μM JQ-1 treatment compared with control condition. NES, normalized enrichment score. (F) Schematic illustration of JQ-1 and radiation treatment paradigm using the *in vivo* xenograft mouse model. (G) Survival analysis of mice with GBO cell transplantation (UP-10311) in different treatment groups: JQ-1 injection followed by radiation (*n* = 5), vehicle injection followed by radiation (*n* = 5), JQ-1 injection alone (*n* = 4), and vehicle injection alone (*n* = 5). Statistical significance is assessed using log rank tests, with multiple-testing correction by Benjamini-Hochberg procedure with *p* values as indicated. See also Figure S4; Table S4.

### Validation of a BET inhibitor in enhancing radiotherapy in the xenograft model

To validate the efficacy of BET inhibitors in enhancing radiotherapy *in vivo*, we performed intracranial transplantation of dissociated GBO cells in immunodeficient mice.^28^ Dissociated GBO cells were used rather than rGBOs because transplantation of intact rGBOs presents technical challenges related to handling and reproducible implantation of small aggregates. Four weeks after tumor transplantation, daily intraperitoneal injections of JQ-1 or vehicle were administered for three consecutive days, followed by a single 8-Gy radiotherapy session delivered via a benchtop irradiator with only the head exposed (Figure 4F). We found that mice pre-treated with JQ-1 followed by radiotherapy survived significantly longer than control mice, while JQ-1 treatment or radiation alone did not significantly extend mouse survival compared with control mice (Figure 4G). Together, these results suggest that targeting BET proteins may offer a promising strategy to improve radiotherapy efficacy.

## Discussion

Studies using *ex vivo* tumor organoids to comprehensively map large-scale heterogeneous treatment responses in GBM remain scarce. Here, we developed a standardized rGBO model and applied it to profile tumor responses to radiotherapy across a large patient cohort (*n* = 48). We demonstrated that rGBO responses significantly correlate with tumor *MGMT* promoter methylation and patient survival. By integrating tumor transcriptomic profiles, we identified molecular signatures associated with differential radiotherapy responses. Leveraging these signatures, we performed *in silico* compound screening, identified BET inhibitors as promising radiosensitizers, and confirmed their efficacy in combination with radiotherapy with subsequent *in vitro* and *in vivo* experiments, providing a proof-of-principle case for our integrated approach. Collectively, these findings underscore the potential of rGBOs as a clinically relevant model for testing personalized treatment efficacy, investigating mechanisms of resistance, and exploring novel therapeutic strategies.

Inter- and intra-tumor heterogeneity contributes significantly to variability in therapeutic responses among cancer patients and remains a key challenge for preclinical modeling.^54^^,^^55^^,^^56^ Compared with traditional cancer models such as cell lines and xenografts,^57^^,^^58^^,^^59^ patient-derived tumor organoids better preserve cellular and molecular diversity while enabling rapid generation,^24^^,^^60^^,^^61^ offering a personalized approach to preclinical modeling.^20^^,^^21^^,^^22^^,^^26^^,^^27^^,^^29^^,^^62^ Previous studies have demonstrated the feasibility of using GBM organoids to model treatment responses.^13^^,^^26^^,^^27^^,^^29^^,^^35^^,^^63^ However, whether such approaches can be applied to systematically characterize heterogeneous radiotherapy responses across large GBM cohorts and identify clinically relevant determinants of radiosensitivity remains unclear. Here, we addressed these questions by developing a standardized rGBO model that minimizes inter-organoid variability and enables systematic investigation of radiotherapy responses across patients. rGBOs retained protein and transcriptomic profiles of their matched GBOs, exhibiting differential radiation responses that significantly correlated with corresponding patient survival. Further validation in larger cohorts is needed to determine the predictive utility of rGBOs for clinical decision-making in radiotherapy. Nonetheless, these results, together with previous studies,^13^^,^^26^^,^^27^^,^^29^^,^^63^ support rGBOs as a faithful model to evaluate treatment strategies and investigate mechanisms underlying differential treatment responses and resistance, offering a scalable and quantitative framework for personalized screening of standard and novel therapeutics across large patient cohorts.

Standard-of-care radiotherapy in GBM patients is highly variable in its efficacy and often limited by tumor resistance.^8^ Few studies have explored mechanisms underlying radiotherapy resistance across large GBM patient cohorts, with most examining only a few cell lines.^64^^,^^65^^,^^66^ Molecularly targeted agents such as EGFR inhibitors and antiangiogenic agents such as bevacizumab can act as radiosensitizers when combined with radiotherapy by modulating cell survival or angiogenesis^67^^,^^68^; however, clinical trials have shown limited efficacy of these combinations in GBM,^68^^,^^69^^,^^70^ highlighting the need for novel therapeutic strategies. To better understand and overcome mechanisms of radiotherapy resistance, we integrated transcriptomic analyses of tumor samples with differential radiation responses of rGBOs. We identified molecular features associated with differential radiotherapy responses, including both canonical and less-characterized pathways associated with resistance. We next established a pipeline for *in silico* compound discovery using perturbation datasets^48^^,^^49^ and identified BET inhibitors as synergistic agents that enhance radiotherapy efficacy in GBM by downregulating radioresistant programs. We further validated their *in vivo* efficacy, demonstrating that combining BET inhibition with radiotherapy significantly improved mouse survival compared with monotherapy. JQ-1 and other BET inhibitors have previously been proposed as therapies for GBM,^52^^,^^71^^,^^72^^,^^73^ potentiating radiosensitivity and synergizing with other treatments for pediatric brain tumor^74^ and other types of cancer.^75^^,^^76^^,^^77^^,^^78^^,^^79^ Independent studies have shown that this class of drugs can sensitize GBM cells to radiation^52^ or chemotherapy^73^ by repressing mesenchymal transition or MGMT expression, respectively, providing independent support for our findings and reinforcing the utility of the rGBO platform in revealing mechanisms of treatment resistance. Using identification of BET inhibitors as a proof-of-principle case, our study highlights the potential of the integrated pipeline, including rGBO generation, treatment modeling, and *in silico* compound discovery targeting broader transcriptomic signatures, for application to other treatment strategies as well as other diseases and therapeutic contexts.

In summary, our study develops a standardized patient tumor-derived organoid platform for quantitative testing at scale, validates the potential of rGBOs to recapitulate patient-specific treatment responses, provides a resource linking rGBO radiotherapy responses to transcriptomic signatures, and identifies candidate agents to potentiate standard-of-care radiotherapy.

### Limitations of the study

While rGBOs represent a promising and innovative platform for assessing treatment responses, this study has several limitations. First, despite efforts to validate the identified molecular signatures using an independent cohort and successful identification of adjuvant therapies, the sample size was constrained by tissue availability and our laboratories’ culture capacity. Consequently, the predictive utility of rGBOs and the identified signatures for clinical decision-making require further validation in larger cohorts. Due to the limited sample size of individual subgroups, findings from stratified analyses by recurrence status or other clinical features should be interpreted with caution. Larger and more balanced cohorts may enable the identification of refined radiosensitivity signatures across distinct GBM subtypes. Second, we deliberately simplified our rGBO model by using established GBOs lacking TME components, as variable TME composition precludes standardized high-throughput analyses and cannot be distinguished by ATP-based viability assays. Notably, almost all tumor organoid models developed for high-throughput applications do not incorporate a TME,^20^^,^^80^ highlighting the technical challenges in this setting. Although the radioresistant signatures include pathways commonly associated with the TME, many are also expressed by malignant GBM cells (Figure S4C), leaving the relative contributions of tumor-intrinsic and microenvironment-derived signals unresolved in the current model. Accordingly, developing organoid platforms that incorporate TME components remains an important future direction. Additionally, rGBOs lack the spatial organization and tissue architecture present in intact GBOs as well as other systemic factors, such as the BBB and the immune system. Consequently, therapeutic effects mediated by these features cannot be adequately assessed using the current rGBO platform. Third, despite efforts to correlate radiation responses in rGBOs with patient genetics and clinical parameters, these factors may not be optimal predictors of treatment response. Future studies could incorporate additional clinical measures, such as longitudinal MRI data, to more accurately assess patient responses to radiotherapy. Last, the limited availability of GBO material restricts the scope of *in vivo* functional validation, resulting in relatively small numbers of animals without bioluminescence live tracking and histological confirmation of tumor burden. Therefore, the *in vivo* efficacy of BET inhibitors combined with radiotherapy should be further validated across a broader range of patient-derived GBOs and glioma lines to strengthen the findings and better elucidate the underlying mechanisms. Future studies are needed to evaluate BET inhibitors beyond JQ-1 *in vivo*, including systematic examination of clinically relevant BET inhibitors with optimized dosing, scheduling, and combination with radiotherapy, to better assess their translational potential for GBM therapy.

## Resource availability

### Lead contact

Further information and requests for resources and reagents should be directed to and will be fulfilled by the lead contact, Hongjun Song (shongjun@pennmedicine.upenn.edu).

### Materials availability

GBOs generated in this study have been deposited in the University of Pennsylvania Brain Tumor Bank. There are restrictions on the availability of biobanked GBOs because of the absence of an external centralized repository and the need to maintain the GBO stock. However, certain GBOs generated in this study will be made available upon reasonable request with the approval of an internal review board and completion of a materials transfer agreement.

### Data and code availability

• The sequencing data reported in this study are deposited, including FASTQ and read count files from RNA-seq of GBOs, rGBOs, and matched primary GBM tissue, at NCBI GEO with accession no. GEO: GSE300985. The transcriptomic signatures associated with radiation responses and the list of compound candidates and their corresponding scores generated using connectivity mapping are provided as Tables S1, S2, S3, and S4.
• This paper does not report custom code.
• Any additional information required to reanalyze the data reported in this paper is available from the lead contact, Hongjun Song, upon request.

## Acknowledgments

We thank the patients and their families for the generous donations of tissue specimens; all members of the Song & Ming laboratories for discussion and suggestions for this study; and Angelina Angelucci, Giana Alepa, Emma LaNoce, and Brian Temsamrit for laboratory support. We thank the Cell and Animal Radiation Core (CARC, RRID: SCR_022377) in the University of Pennsylvania for the radiotherapy services. This work was supported by the 10.13039/100000002National Institutes of Health (R35NS116843 to H.S., R35NS097370 and R35NS137480 to G-l.M., and R01CA304417 to D.M.O. and H.S.), Dr. Miriam and Sheldon G. Adelson Medical Research Foundation (to G-l.M., R.K., and D.H.G.), the Abramson Cancer Center Glioblastoma Translational Center of Excellence (to H.S., Z.A.B., and D.M.O.), The Dean’s Innovation Fund (to H.S.), The Institute for Regenerative Medicine and Department of Neurosurgery at University of Pennsylvania (to H.S., D.M.O., and M.P.N.), the Templeton Family Initiative in Neuro-Oncology (to Z.A.B. and D.M.O.), and the Maria and Gabriele Troiano Brain Cancer Immunotherapy Fund (to Z.A.B. and D.M.O.).

## Author contributions

X.W., Y.S., D.Y.Z., K.H.P., Y.F., W.D., and Z.Z. contributed to GBO generation and culture. X.W. performed rGBO radiotherapy assessments, immunohistochemistry, RNA-seq library preparation, compound screening, and *ex vivo* synergy experiments. Y.S. contributed to *in silico* compound identification, analysis of sequencing data, and clinical data collection. P.S., R.S., X.W., and Y.S. performed *in vivo* radiation studies in mice. J.F.D. and Y.F. guided *in vivo* radiotherapy procedures. S.J.B., M.P.N., E.L.-L.P., and M.A.G. performed neuropathological analysis of tumors and GBOs. D.M.O., N.M.A., Z.A.B., M.P.N., E.L.-L.P., M.A.G., and R.C. contributed to tumor tissue collection. X.W., Y.S., G.-l.M., and H.S. conceived the project and wrote the manuscript with inputs from all authors.

## Declaration of interests

The authors declare no competing interests.

## STAR★Methods

### Key resources table

**Table:** 

REAGENT or RESOURCE	SOURCE	IDENTIFIER
**Antibodies**		

Goat anti-Doublecortin	Santa Cruz Biotechnology	Cat# sc-8066; RRID: AB_2088494
Goat anti-Olig2	R&D Systems	Cat# AF2418; RRID: AB_2157554
Mouse anti-GFAP	Sigma-Aldrich	Cat# MAB360; RRID: AB_11212597
Mouse Anti-Ki-67	BD Biosciences	Cat# 550609; RRID: AB_39377
Mouse anti-SOX9	Sigma-Aldrich	Cat# AF146; RRID: AB_2239761
Rabbit anti-EGFRvIII	Cell Signaling	Cat# 64952 S; RRID: AB_2773018
Rabbit anti-HOPX	Santa Cruz Biotechnology	Cat# sc-30216; RRID: AB_2120833
Rat anti-Tenascin C	R&D Systems	Cat# MAB2138; RRID: AB_2203818
donkey anti-goat IgG Alexa Fluor 488	Thermo Fisher	Cat# A-11055; RRID: AB_2336933
donkey anti-goat IgG Alexa Fluor 647	Thermo Fisher	Cat# A-21447; RRID: AB_2535864
donkey anti-rabbit IgG Alexa Fluor 555	Thermo Fisher	Cat# A-31572; RRID: AB_162543
donkey anti-mouse IgG Alexa Fluor 488	Thermo Fisher	Cat# A-21202; RRID: AB_141607
donkey anti-mouse IgG Alexa Fluor 555	Thermo Fisher	Cat# A-31570; RRID: AB_2536180
donkey anti-mouse IgG Alexa Fluor 647	Thermo Fisher	Cat# A-31571; RRID: AB_162542

**Chemicals, peptides, and recombinant proteins**		

1× RBC lysis buffer	Thermo Fisher Scientific	Cat# 00433357
2-Mercaptoethanol	Thermo Fisher Scientific	Cat# 21985023
ABBV-744	MedChemExpress	Cat# HY-112090
Antibiotic-Antimycotic (100×)	Gibco	Cat# 15240062
AZD-5069	MedChemExpress	Cat# HY-19855
B27 supplement without vitamin A	Gibco	Cat# 12587010
BGT226	MedChemExpress	Cat# HY-13334 A
BI-2536	MedChemExpress	Cat# HY-50698
Bovine serum albumin (BSA)	Sigma-Aldrich	Cat# B6917; CAS# 9048–46–8
DAPI	Sigma-Aldrich	Cat# 10236276001; CAS# 28718–90–3
Dimethyl sulfoxide (DMSO)	Sigma-Aldrich	Cat# D2650; CAS# 67–68–5
DMEM/F12 Medium	Gibco	Cat# 11330032
Donkey Serum	Sigma-Aldrich	Cat# S30-100ML
Dulbecco’s phosphate-buffered saline (DPBS), no calcium, no magnesium	Thermo Fisher Scientific	Cat# 14190144
Formaldehyde, 16%, methanol free, Ultra-pure	Polysciences	Cat# 18814–10; CAS#: 50–00–0
Foscarnet	MedChemExpress	Cat# HY-B1318
Geldanamycin	MedChemExpress	Cat# HY-15230
GlutaMAX™	Gibco	Cat# 35050038
GSK778	MedChemExpress	Cat# HY-136570
Hibernate™-A Medium	Gibco	Cat# A1247501
I-BET151	MedChemExpress	Cat# HY-13235
Insulin solution human	Sigma-Aldrich	Cat# I9278
Ipatasertib	MedChemExpress	Cat# HY-15186
JQ-1	MedChemExpress	Cat# HY-13030
KAPA SYBR FAST 2× MasterMix	Thermo Fisher Scientific	Cat# 50–196–5206
Masitinib	MedChemExpress	Cat# HY-10209
Minimum Essential Medium from non-essential amino acids	Gibco	Cat# 11140050
N2 supplement	Gibco	Cat# 7502048
Neurobasal Medium	Gibco	Cat# 11570556
Nuclease-Free Water (not DEPC-Treated)	Thermo Fisher Scientific	Cat# AM9937
Paxalisib	Selleckchem	Cat# S8163
Penicillin-Streptomycin (5,000 U/mL)	Thermo Fisher Scientific	Cat# 15070063
PFI-1	MedChemExpress	Cat# HY-16586
RNA Clean & Concentrator 5	Zymo Research	Cat# R1013
SB225002	MedChemExpress	Cat# HY-16711
SMARTScribe Reverse Transcriptase	Takara	Cat# 639537
SPRIselect Reagent	Beckman Coulter	Cat# B23318
Tanespimycin	MedChemExpress	Cat# HY-10211
Triton™ X-100	Sigma-Aldrich	Cat# T9284
Torin-2	MedChemExpress	Cat# HY-13002
Tris (1 M), pH 8.0, RNase-free	Thermo Fisher Scientific	Cat# AM9855G
TRIzol reagent	Thermo Fisher Scientific	Cat# 15596026
Trypan blue stain, 0.4%	Thermo Fisher Scientific	Cat# T10282
Tween® 20	Sigma-Aldrich	Cat# P9416-50mL; CAS# 9005–64–5
VECTASHIELD Vibrance Antifade Mounting Medium	Vector Laboratories	Cat# H170010
XMD8-92	Selleckchem	Cat# S7525

**Critical commercial assays**		

Bioanalyzer high sensitivity DNA analysis	Agilent	Cat# 5067–4626
Brain Tumor Dissociation Kit	Miltenyi Biotech	Cat# 130–095–942
CellTiter-Glo® 3D Cell Viability Assay	Promega	Cat# G9683
KAPA Library Quantification Kit for Illumina NGS	Kapa Biosystems	Cat# KK4835
NEBNext® Poly(A) mRNA Magnetic Isolation Module	NEB	Cat# E7490L
NEBNext® Ultra™ II RNA Library Prep Kit for Illumina®	NEB	Cat# E7775L
Qubit dsDNA HS Assay Kit	Thermo Fisher Scientific	Cat# Q33231

**Deposited data**		

RNA sequencing of primary, GBO, and reaggregate GBOs	This paper	NCBI GEO Accession GEO: GSE300985

**Experimental models: Cell lines**		

Glioblastoma organoid samples	This paper	Table S1

**Experimental models: Organisms/strains**		

NU/J *Mus musculus*, female	The Jackson Laboratory	Cat# 002019; RRID: IMSR_JAX:002019

**Software and algorithms**		

Adobe Illustrator 28.2 64-bit	Adobe	https://www.adobe.com/products/illustrator.html; RRID: SCR_010279
bcl2fastq v.2.19.0.316	Illumina	https://support.illumina.com/sequencing/sequencing_software/bcl2fastq-conversion-software.html; RRID:SCR_015058
DESeq2 v.1.22.2	Love et al.^41^	https://bioconductor.org/packages/release/bioc/html/DESeq2.html; RRID:SCR_015687
GRCh38 v 28	Genome Reference Consortium	https://www.ncbi.nlm.nih.gov/assembly/GCF_000001405.38
ImageJ	Open source	https://ImageJ.net/ij/; RRID: SCR_003070
R Project v3.6.0	Open source	https://www.r-project.org/; RRID: SCR_001905
RStudio v1.2.1335	Open source	https://rstudio.com/; RRID: SCR_000432
RSEM v1.3.3	Li et al.^81^	https://github.com/deweylab/RSEM; RRID:SCR_000262
STAR v.2.6.1d	Dobin et al.^82^	https://github.com/alexdobin/STAR; RRID:SCR_015899
Zen Blue 3.5	Zeiss	RRID:SCR_013672

**Other**		

#1.5 glass coverslip	Fisher Scientific	Cat#12–544-EP
Bioanalyzer 2100	Agilent	Cat# G2939BA
Cabinet X-ray irradiator	Precision X-ray	Cat# X-RAD320ix
Cell counting slides	Thermo Fisher Scientific	Cat# C10228
Clear TC-treated 6-Well Plates	Corning	Cat# 3506
CO_2_ Resistant Shakers	Thermo Fisher Scientific	Cat# 88881101
Countess II Automated Cell Counter	Thermo Fisher Scientific	Cat# AMQAX1000
Cryostat	Leica Biosystems	Cat# CM3050
Disposable Petri Dishes	VWR	Cat# 25384–088
DynaMag™-2 Magnet	Thermo Fisher Scientific	Cat# 12321D
ImmEdge® Hydrophobic Barrier PAP Pen	Vector Laboratories	Cat# H-4000
MACS® SmartStrainer, 70 μm	Miltenyi Biotec	Cat#130–098–462
NanoDrop 2000	Thermo Fisher Scientific	Cat# ND-2000
Plate Reader	Tecan	Cat# 30050303
Qubit 3 Fluorimeter	Thermo Fisher Scientific	Cat# Q33216
Slide Moisture Chamber	Newcomer Supply	Cat# 68432 A
StepOnePlus™ Real-Time PCR System	Thermo Fisher Scientific	Cat# 4376600
Superfrost™ Plus Gold Microscope Slides	Fisher Scientific	Cat# 15–188–48
T100™ Thermal Cycler	Bio-Rad	Cat# 1861096EDU
Thermowell™ PCR tube strips	Corning	Cat# 6547
Tissue Freezing Medium	General Data	Cat# TFM-C
Tissue-Tek® Cryomold®	Electron Microscopy Sciences	Cat# 62534–10
Tube rotator	VWR	Cat# 10136–084
Vannas Spring Scissors – 3 mm cutting edge	Fine Science Tools	Cat# 15000–10

### Experimental model and study participant details

#### Human subjects

Human brain tumor tissue was collected at the Hospital of the University of Pennsylvania under a protocol (#816686) approved by the University of Pennsylvania’s Institutional Review Board after informed patient consent. Patient tumor samples were de-identified and used following technical and ethical guidelines for biomedical research. A total of 48 patient cases from both males and females were included in this study (Table S1).

#### Animals

All procedures related with animals in this study were performed following the protocol (#806246) approved by the Institutional Animal Care and Use Committee (IACUC) at the University of Pennsylvania. Mice were housed in groups of up to five per cage, under a 14-h light/10-h dark cycle, with access to food and water *ad libitum*. Female athymic nude (NU/J) mice, aged 4–8 weeks, were used for all experiments. Animals were monitored at least twice weekly for weight loss and were routinely checked for physical or neurological abnormalities.

### Method details

#### Collection of patient GBM samples and generation of GBOs

For generation of GBOs, we followed our previously published protocols.^13^^,^^83^ Briefly, fresh human brain tumor tissue was collected and stored in cold Hibernate A media containing 1× GlutaMAX and 1× Antibiotic-Antimycotic. Tissue was dissected into 1–2 mm^3^ pieces under a dissection microscope in a culture hood. Tissue pieces were then washed by DPBS and incubated with cold RBC lysis buffer to remove red blood cells followed by DPBS wash. Tissue pieces were transferred to 6 well plates in GBO culture media containing 50% DMEM:F12, 50% Neurobasal, 1× Penicillin-Streptomycin, 1× NEAAs, 1× GlutaMax, 1× B27 w/o vitamin A supplement, 1× N2 supplement, 1X 2-mercaptoethanol, and 2.5 μg/mL human insulin, and cultured using an orbital shaker with a speed of 110 rpm in a cell culture incubator with 37°C, 5% CO_2_ and 85% humidity. Media was changed every two days. Successful GBO generation was marked by a round smooth rim of the organoids, clear color and dense texture.

#### GBO digestion and reaggregation

GBOs were digested using a brain tumor tissue dissociation kit (Miltenyi Biotech, 130–0950929) following the manufacturer’s instructions. 5–10 GBOs were transferred to a 1.5 mL Eppendorf tube and washed by DPBS, 1 mL digestion mix was added. The mixture was incubated with rotation in 37°C for 30 min. Occasional pipetting was used to help digestion. Samples were centrifuged at 400 g for 3 min at 4°C and resuspended using GBO culture media. Cell viability was checked using trypan blue staining and an automatic cell counter. A desired viability rate would be >60%. Cells were then seeded in 96 well U-bottom plates with a final concentration of 5000 cells in 50 μl per well. They were cultured under static culture conditions in a normal cell culture incubator. Cells would reaggregate into a small organoid at the well bottom overnight and remain in the same plate for subsequent treatments or assays.

#### Sample processing, immunohistochemistry and imaging

GBOs were fixed with 4% formaldehyde for 1 h at room temperature with rotation. After triple washing with DPBS, GBOs were transferred to 30% sucrose at 4°C overnight until processing. GBOs were sectioned into 20 μm slices using a cryostat and mounted onto charged slides. Slides were stored at −20°C until processing. For immunohistochemistry, the sections were outlined with a hydrophobic pen and washed with TBS containing 0.1% Tween 20 (v/v) (TBST). Permeabilization and blocking were done with a blocking buffer containing TBST with 10% donkey serum (v/v), 0.5% Triton X-100 (v/v), and 1% BSA (w/v) for 1 h at room temperature. Sections were next incubated overnight at 4°C with primary antibodies (see key resources table) diluted 1:500 in antibody dilution buffer (TBST, 5% donkey serum, 0.1% Triton X-100). After triple washing with TBST, sections were incubated with secondary antibodies (see key resources table) diluted 1:1000 in antibody dilution buffer for 1–2 h at room temperature. After triple washing with TBST, slides were mounted in mounting solution, covered with coverslips, and sealed with nail polish.

For whole-mount immunohistochemistry of rGBOs, rGBOs were transferred to U-bottom 96-well plates and fixed with 4% formaldehyde for 1 h at room temperature. After triple washing with DPBS, rGBOs were blocked with the blocking buffer for 1 h at room temperature and then processed using the same procedures outlined above. During mounting, a circle of nail polish was applied to the glass to create a raised edge, and once dry, rGBOs were placed inside the circle. After removing excess buffer, the rGBOs were mounted in mounting solution, covered with coverslips, and sealed with nail polish.

GBO sections and rGBOs were imaged as z-stacks using a Zeiss LSM 810 or Zeiss LSM 710 confocal microscope (Zeiss) with a 20× objective and Zen 2 software (Zeiss). Image analysis was performed using ImageJ software.

#### RNA-seq library preparation and analysis

For RNA-seq, paired primary GBM tissue, GBO and rGBO samples (Figure 2) were snap-frozen on dry ice and stored at −80°C until processing. The samples were homogenized in TRIzol using a disposable pestle and handheld motor. RNA purification was performed with a Zymo RNA Clean & Concentrator kit after phase separation following the manufacturer’s instructions. RNA concentration and quality were measured using a Nanodrop 2000. RNA sequencing libraries were prepared using the NEBNext Poly(A) mRNA Magnetic Isolation Module to extract mRNA, followed by library preparation with the NEBNext Ultra II RNA Library Prep Kit for Illumina following the manufacturer’s instructions. Library size, concentration and quality were assessed using the Qubit dsDNA assay and Bioanalyzer. Sequencing of samples was performed on either a NovaSeq 6000 (Illumina) or a NextSeq 550 (Illumina) with a final sequencing depth of approximately 5–10 million reads per sample.

Raw sequencing data were demultiplexed using bcl2fastq2 v.2.19.0.316 (Illumina) with adaptor trimming disabled. Alignment was carried out with STAR v.2.6.1d^82^ We used GRCh38 (gencode) as the reference genome and gencode v.28 GTF for the annotation file. Reads were quantified at the exon level before being summarized as gene counts using RSEM v1.3.3.^81^ Subsequent analyses were performed in R v.3.6.0 using RStudio v1.2.1335. DEG analysis was carried out using the DESeq2 analysis suite,^41^ where the full model design was ∼mean_auc + Sample + *MGMT* + Recurrence + Batch (sequencing batch) to enable identification of genes whose expression levels significantly correlate with treatment response indicated by mean_auc. A patient term was not included because mean_auc was defined at the patient level. Effect size estimation was computed with apeglm,^84^ and statistical significance was assessed with a Benjamini-Hochberg corrected *p*-value <0.05. Gene ontology enrichment was performed using ClusterProfiler.^85^

For RNA-seq of GBOs after treatment of JQ-1 (Figure 4), to enable increased sample size and throughput, we leveraged our Smart-seq3 based platform.^28^ GBOs were reaggregated into rGBOs of ∼5000 cells in 96-well U-bottom plates overnight and treated for 3 days in concentrations of JQ-1. They were then taken for sample lysis, library preparation, and data preprocessing as described.^28^ Differential gene expression and GO enrichment analyses were performed as described above but with the model design ∼Patient + Condition. We used the AddModuleScore function from Seurat v5^86^ to assess changes in the radioresistant gene signature (Tables S2B and S2C) across JQ-1 conditions from different patients.

#### Single cell analysis and comparison between GBOs and rGBOs

To assess concordance between GBOs and rGBOs, we performed scRNA-seq via our Smart-seq3-based platform as previously described.^28^^,^^87^ Smart-seq3 scRNA-seq data of GBOs were obtained from GEO: GSE274460,^28^ and matched rGBOs were generated as described above, with sample lysis, library preparation, and sequencing as previously described.^28^ We performed downstream analysis with the Seurat v5 framework and integrated GBO and rGBO data via the *IntegrateLayers* function with CCA integration.^86^ CCA clusters were then annotated based on marker gene expression prior to visualization of cluster distributions.

#### Radiation and *in silico* compound screening in rGBOs or GBOs

Different doses of radiation (2, 4, 8, 10 or 20 Gy in dose-finding experiments; 0, 2, 4, 8 Gy in subsequent experiments) were applied to rGBOs at a dose rate of 2.65 Gy/min using a Precision XRAD320iX cabinet irradiator. We recommend using at least 3 rGBO replicates per treatment condition, as rGBOs reduce inter-organoid variability and provide consistent viability readouts. In this study, a 4-day post-radiation endpoint was used, which balances limited media volume with sufficient time to observe an effective radiation response; generally, experiments should be conducted within several days to maintain optimal media conditions, and endpoints may be adjusted depending on the treatment type and readout assay. The specific radiation dose should be determined based on experimental goals and tumor type, with preliminary testing to identify an effective range. In our experiments, 8 Gy was chosen as it achieved a plateau in response within the 4-day observation window. After 4 days, cell mass was assessed using the CellTiter-Glo 3D Cell Viability Assay and a plate reader, following the manufacturer’s instructions.

Raw luminescence values were normalized to the mean luminescence of the control group (0 Gy) within each patient and experimental batch to determine fold changes in viability. Replicate patients across multiple technical batches were consolidated by calculating the mean fold change per dose. To quantify the cumulative dose-response for each patient, the area under the curve (AUC) metric was calculated via the trapezoidal rule with the bayestestR package,^88^ and a higher AUC indicates greater radiation resistance.

For compound treatment in rGBOs, different compounds with variable concentrations or DMSO were added to the culture media with at least three replicates. Different doses of radiation (0, 2, 4, or 8 Gy) were applied to rGBOs using the Precision XRAD320iX cabinet irradiator. The cell viability was measured as above. The ZIP synergy score was calculated by comparing the observed luminescence values (normalized to the no-treatment condition) with the expected values under the assumption of zero interaction and averaging the differences across all dose combinations.

For JQ-1 and radiation treatment of whole GBOs, JQ-1 at varying concentrations or DMSO was added to the culture medium. The following day, GBOs received 8 Gy radiation or mock treatment. Images of individual GBOs were taken before and after the treatment using a brightfield microscope and Zen software. The 2D projected area of each GBO was quantified in ImageJ by manually outlining each GBO and measuring the area within the outlined region. The synergy score was calculated based on quantified area and normalized to the control condition without JQ-1 treatment or radiation.

#### Intracranial transplantation of GBO cells and *in vivo* JQ-1 and radiation treatment

Intracranial transplantation of GBO cells was performed in female adult athymic nude mice (The Jackson Laboratory). GBO (UP-10311) single-cell suspension was prepared using the digestion method described above. Mice were anesthetized with 2% isoflurane in 100% oxygen. Anesthesia depth was confirmed by toe pinch, and ophthalmic ointment was applied to the eyes to prevent dryness. The incision site was disinfected with Betadine solution, followed by a subcutaneous injection of the local anesthetic bupivacaine. A midline scalp incision (∼0.5–1.5 cm) was made, and a hole was drilled over the target region using a micromotor drill. A Hamilton syringe with a 26-gauge needle was used to inject 100,000 tumor cells in 2 μL of Hibernate A media at a flow rate of 0.5 μL/min. After the injection, the incision was sutured, and Meloxicam was administered locally following surgery and once every 24 h for the next 48 h. Mice were placed on a 37°C warming pad until they regained the ability to move freely before being returned to their home cages. Post-surgery mice were monitored daily for the first 3 days and then twice weekly to assess recovery, weight loss (≥15% reduction), or any signs of physical abnormalities.

After 4 weeks, mice were randomly assigned to one of four groups: JQ-1 injection followed by radiation, vehicle injection followed by radiation, JQ-1 injection alone, and vehicle injection alone. JQ-1 was dissolved in DMSO to a concentration of 50 mg/mL and stored at −20°C. A fresh JQ-1 solution was prepared daily by mixing 9 parts of 10% beta-cyclodextrin with 1 part of JQ-1 stock, yielding a final concentration of 5 mg/mL. Mice in the JQ-1 injection alone and JQ-1 injection followed by radiation groups received daily intraperitoneal injections of JQ-1 (20 mg/kg in 10% beta-cyclodextrin) for 3 consecutive days. Mice in the vehicle injection alone and radiation with vehicle injection groups received daily intraperitoneal injections of vehicle (10% DMSO in 10% beta-cyclodextrin). On the following day, mice in the radiation alone and combination treatment groups were anesthetized with an intraperitoneal injection of a ketamine cocktail (0.003 mL/g body weight; containing 20 mg/mL ketamine, 3 mg/mL xylazine, and 0.25 mg/mL acepromazine). Anesthesia depth was confirmed by toe pinch, and ophthalmic ointment was applied to the eyes. Mice were then subjected to 8-Gy radiation using a Precision XRAD320iX cabinet irradiator, with a lead shield (6 mm thickness) covering the body to allow exposure to the head only. Mice were weighed and checked twice weekly to assess overall health.

### Quantification and statistical analysis

The details regarding sample sizes and statistical analyses are provided in the figure legends or the main text. The “*n*” value indicates the number of technical replicates of rGBOs, GBOs, patients, or mice used in the experiments. A *p*-value of less than 0.05 was considered statistically significant. GBO size quantifications, brightfield images were analyzed, and outlines were traced using ImageJ. Cell counting was conducted with the ImageJ Cell Counter plugin. All plots were generated with the ggplot2 package, with individual data points represented by dots. Boxplots show the median and interquartile range with whiskers extending to maximum and minimum values. Bar plots represent mean values, with error bars indicating the standard deviation. All statistical analyses were performed in R using the ggpubr package. The statistical significance of *in vivo* Kaplan-Meier survival curves was determined using the log rank test in Graphpad Prism 10. For patient survival analysis (Figure 1G; Figures S2B and S2C), we only included patients surviving past 100 days to account for the typical time frame of radiotherapy post-surgery.^7^^,^^89^ Cutoffs were determined either using maximally selected rank statistics (optimal cutoff)^90^ or by stratifying patients based on the top and bottom tertiles of the AUC values. To determine the independent prognostic value of the rGBO radiation response, a multivariable Cox proportional hazards model was constructed, and the model included MGMT methylation status, recurrence status, and TP53 mutation status as covariates alongside the mean AUC. Hazard ratios and 95% confidence intervals were visualized via forest plot. Linear mixed-effects modeling was performed with the nlme package in R. Other statistical analyses were indicated in the figure legends.

## Supplemental information

Document S1. Figures S1–S4

Table S1. Patient information, related to Figure 1 Patient information and survival time post-surgery. The dead column indicates whether a patient death was confirmed (1), or if the patient was either lost to follow-up or still alive (0).

Table S2. Transcriptomic signatures associated with radiotherapy responses in rGBOs, related to Figure 2 (A) RNA-seq samplesheet and AUC values. (B) Radioresistant signature: DEGs positively correlated with the mean AUC values of radiotherapy responses in rGBOs. (C) Radiosensitive signature: DEGs negatively correlated with the mean AUC values of radiotherapy responses in rGBOs.

Table S3. Compounds identified by perturbation datasets, related to Figure 3 (A) Compound names and their efficacy scores. (B) Summary of final compound list. (C) Raw cell viability values for candidate compounds with radiotherapy. (D) Raw cell viability values for JQ-1 with radiotherapy. (E) Raw cell viability values for additional BET inhibitors with radiotherapy.

Table S4. DEGs associated with JQ-1 treatment, related to Figure 4 (A) List of DEGs between 0.1 μM JQ-1 treatment and DMSO. (B) List of DEGs between 1 μM JQ-1 treatment and DMSO.

Document S2. Article plus supplemental information
