# Supplementary material for: A standardized patient-derived glioblastoma organoid platform for evaluating radiotherapy efficacy and overcoming resistance
Source: Cell Rep Med. 2026 Oct 7;7(10):103095. doi: 10.1016/j.xcrm.2026.103095 (PMC13710385; doi:10.1016/j.xcrm.2026.103095)
Supplement: Supplementary file 1 [file mmc1.pdf]

Cell Reports Medicine, Volume *7*

Supplemental information

A standardized patient-derived glioblastoma organoid platform for evaluating radiotherapy efficacy and overcoming resistance

Xin Wang, Yusha Sun, Prithvi Sinha, Ruiying Su, Daniel Y. Zhang, Kristen H. Park, Yao Fu, Weifan Dong, Zhijian Zhang, Qing Wang, Daniel H. Geschwind, Riki Kawaguchi, Emily Ling-Lin Pai, Maria A. Gubbiotti, Robert Colbourn, Nduka M. Amankulor, Jay F. Dorsey, MacLean P. Nasrallah, Stephen J. Bagley, Zev A. Binder, Donald M. O'Rourke, Yi Fan, Guo-li Ming, and Hongjun Song

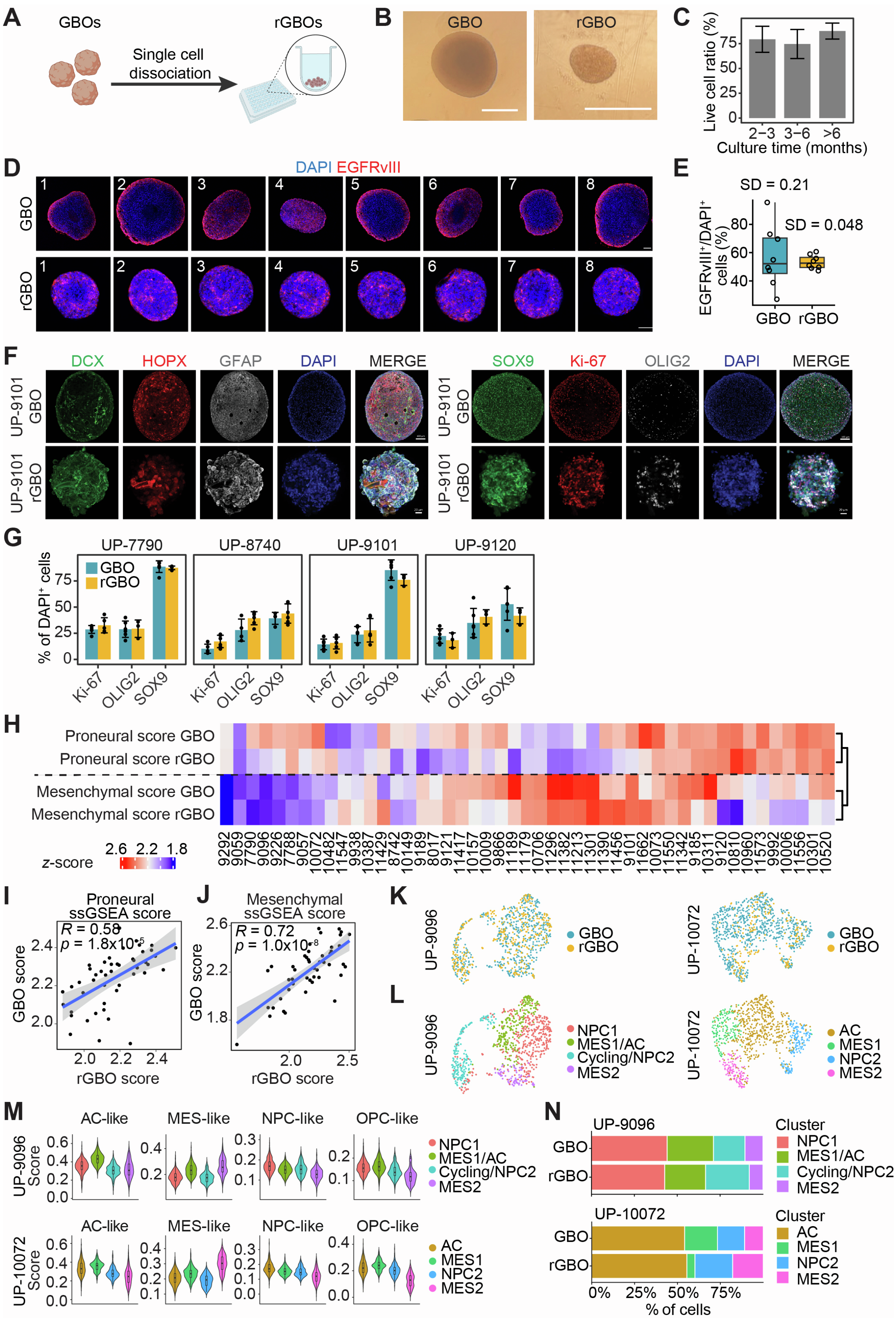
A
GBOs
Single cell dissociation
rGBOs
B
GBO
rGBO
C
Live cell ratio (%)
75
50
25
0
2−3
3−6
>6
Culture time (months)
D
DAPI EGFRvIII
GBO
rGBO
1
2
3
4
5
6
7
8
E
SD = 0.21
SD = 0.048
EGFRvIII⁺/DAPI⁺ cells (%)
80
60
40
GBO
rGBO
F
DCX
HOPX
GFAP
DAPI
MERGE
SOX9
Ki-67
OLIG2
UP-9101 GBO
UP-9101 rGBO
G
UP-7790
UP-8740
UP-9101
UP-9120
% of DAPI⁺ cells
GBO
rGBO
Ki-67
OLIG2
SOX9
H
Proneural score GBO
Proneural score rGBO
Mesenchymal score GBO
Mesenchymal score rGBO
z-score
2.6
2.2
1.8
I
Proneural ssGSEA score
R = 0.58
p = 1.8x10⁻⁵
GBO score
rGBO score
J
Mesenchymal ssGSEA score
R = 0.72
p = 1.0x10⁻⁸
K
UP-9096
UP-10072
GBO
rGBO
L
NPC1
MES1/AC
Cycling/NPC2
MES2
AC
MES1
NPC2
M
AC-like
MES-like
NPC-like
OPC-like
UP-9096 Score
UP-10072 Score
N
Cluster
% of cells
0%
25%
50%
75%

Figure S1. rGBOs preserve molecular features and reduce inter-organoid variability, related to Figure 1.
(A) Schematic illustration of GBO digestion and reaggregation.
(B) Sample bright-field images of a GBO and a matched rGBO of 5000 cells. Scale bars, 500 µm.
(C) Quantification of live cell ratios after digestion in GBOs, grouped by culture time. Statistical significance was assessed using one-way ANOVA ($P = 0.25$).
(D-E) Sample images (**D**) and quantification (**E**) of EGFRvIII-positive cells in individual GBOs and matched rGBOs at 4 days post-reaggregation ($n = 8$ replicates per condition). Boxplots show the median and interquartile range with whiskers extending to maximum and minimum values; points denote individual samples. Scale bars: 100 µm (upper panels), 50 µm (lower panels).
(F-G) Sample images (**F**) and quantification (**G**) of tumor subtype markers in GBOs and matched rGBOs. Scale bars: 100 µm (upper panels), 20 µm (lower panels). All bar plots are plotted as mean ± s.e.m, and each dot represents one GBO ($n \geqslant 3$).
(H) Heatmap showing the clustering of transcriptomic signature scores between GBOs and matched rGBOs ($n = 47$ patients). Patient IDs are abbreviated by omitting the 'UP-' prefix for clarity.
(I-J) Correlation analysis between proneural (**I**) or mesenchymal ssGSEA scores (**J**) of GBOs and the corresponding ssGSEA scores of matched rGBOs ($n = 47$ patients).
(K-L) Uniform manifold approximation and projection (UMAP) plots of single-cell datasets comparing rGBO and GBO cell states with clusters assigned by sample type (**K**) or cell transcriptomic states (**L**). NPC1, neural progenitor cell-like state 1; NPC2, neural progenitor cell-like state 2; MES1, mesenchymal state-like 1; MES2, mesenchymal state-like 2; AC, astrocyte-like state. rGBOs are sampled at 4 days post-reaggregation.
(M) Violin plots showing the distribution of module scores[39] for cellular states (AC-like, MES-like, NPC-like, OPC-like) as described by Neftel *et al.*[1]
(N) Stacked bar plot summarizing the percentages of cells assigned to each cell state in GBOs and matched rGBOs, corresponding to panel **L**.

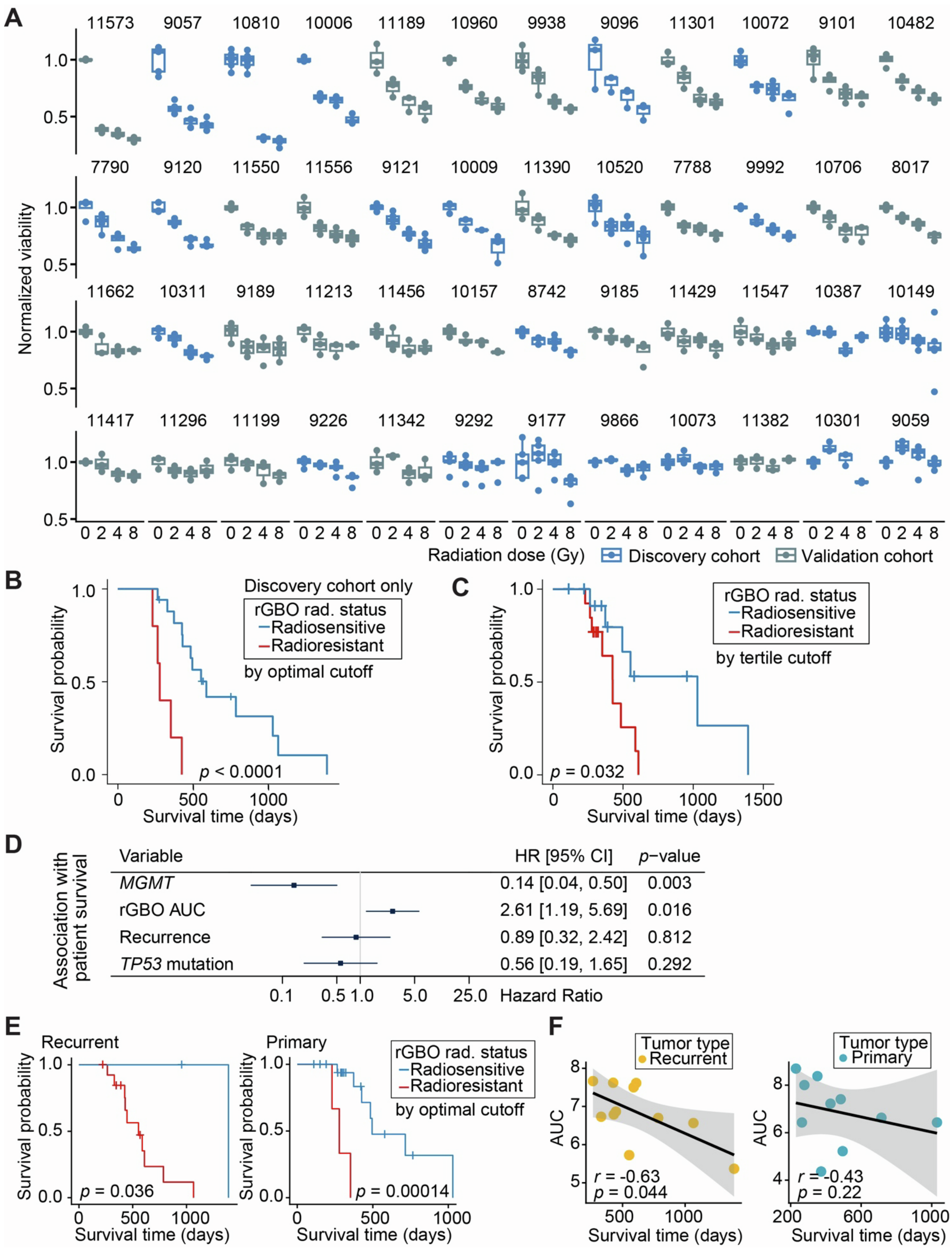
A
11573
9057
10810
10006
11189
10960
9938
9096
11301
10072
9101
10482
7790
9120
11550
11556
9121
10009
11390
10520
7788
9992
10706
8017
11662
10311
9189
11213
11456
10157
8742
9185
11429
11547
10387
10149
11417
11296
11199
9226
11342
9292
9177
9866
10073
11382
10301
9059
Normalized viability
Radiation dose (Gy)
Discovery cohort
Validation cohort
B
Discovery cohort only
rGBO rad. status
Radiosensitive
Radioresistant
by optimal cutoff
p < 0.0001
Survival probability
Survival time (days)
C
rGBO rad. status
Radiosensitive
Radioresistant
by tertile cutoff
p = 0.032
Survival probability
Survival time (days)
D
Association with patient survival
Variable
HR [95% CI]
p-value
MGMT
0.14 [0.04, 0.50]
0.003
rGBO AUC
2.61 [1.19, 5.69]
0.016
Recurrence
0.89 [0.32, 2.42]
0.812
TP53 mutation
0.56 [0.19, 1.65]
0.292
Hazard Ratio
E
Recurrent
p = 0.036
Primary
rGBO rad. status
Radiosensitive
Radioresistant
by optimal cutoff
p = 0.00014
Survival probability
Survival time (days)
F
Tumor type
Recurrent
r = -0.63
p = 0.044
Tumor type
Primary
r = -0.43
p = 0.22
AUC
Survival time (days)

Figure S2. Differential responses to radiotherapy in rGBOs, related to Figure 1.
(A) Normalized cell viability of rGBOs treated with different doses of radiotherapy (0, 2, 4, and 8 Gy) at 6 days post-treatment (n = 48).
(B) Kaplan-Meier survival curve for GBM patients after surgery from discovery cohort (n = 19), stratified by radiotherapy sensitivity in rGBOs. The optimal cutoff was determined using maximally selected rank statistics.
(C) Kaplan-Meier survival curve for patients with GBM after surgery from both cohorts (n = 38 with survival time cutoff of over 100 days), stratified by radiotherapy sensitivity in rGBOs, classified based on the top or bottom tertiles of AUC values as compared to using optimal cutoff in **Figure 1G**. Statistical significance of survival was assessed using the log-rank test.
(D) Multivariable Cox regression analysis of factors associated with overall survival after adjustment for covariates. HR, hazard ratio; CI, confidence interval.
(E) Kaplan-Meier survival curves of GBM patients stratified by radiotherapy sensitivity of rGBOs, shown separately for primary and recurrent tumors. Optimal cutoffs are determined using maximally selected rank statistics. Statistical significance is assessed with log-rank tests.
(F) Spearman correlation between patient survival time and radiation responses of matched rGBOs, analyzed separately for primary and recurrent tumors.

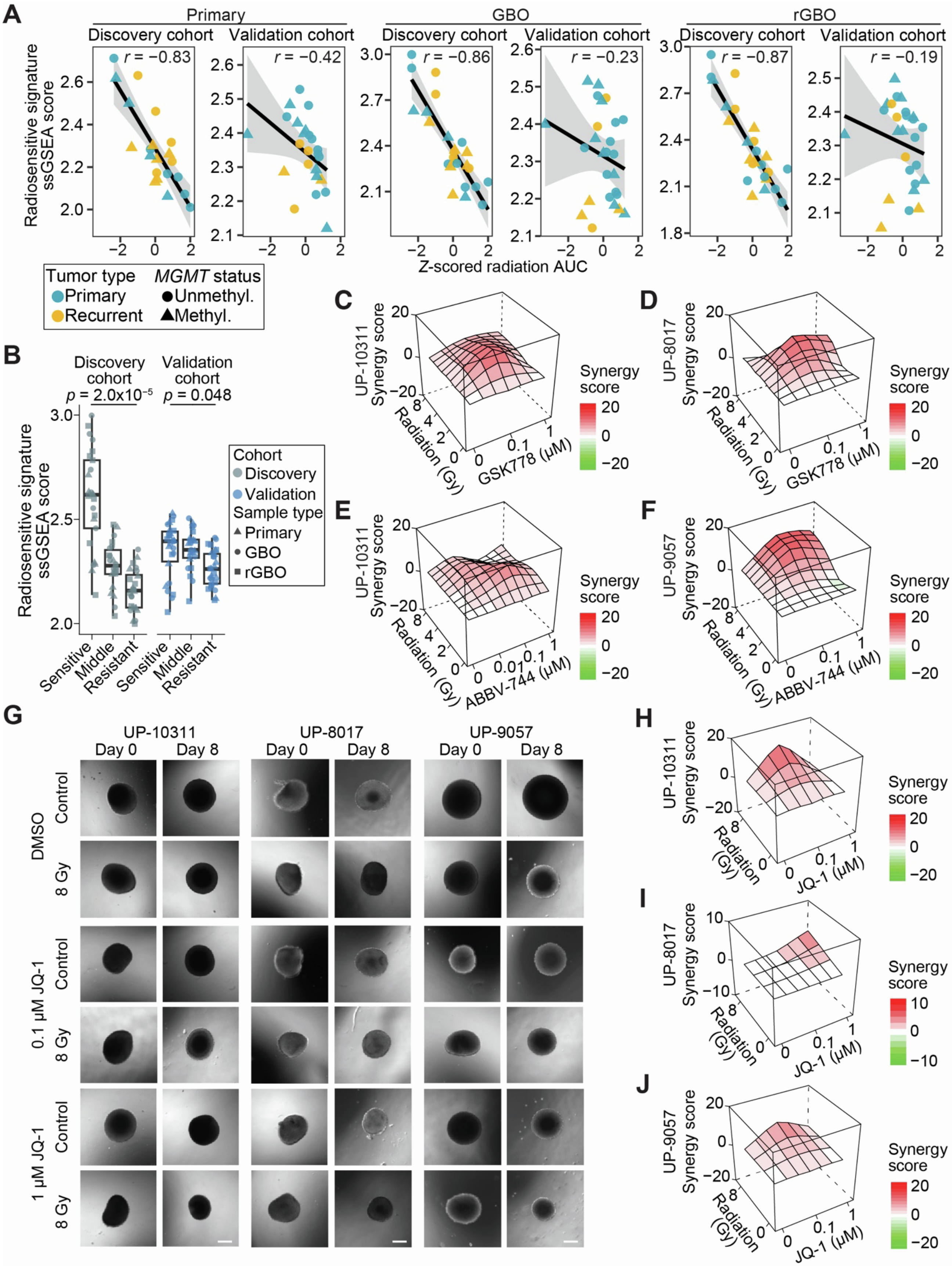
A
Primary
GBO
rGBO
Discovery cohort
Validation cohort
r = −0.83
r = −0.42
r = −0.86
r = −0.23
r = −0.87
r = −0.19
Radiosensitive signature ssGSEA score
Z-scored radiation AUC
Tumor type
Primary
Recurrent
MGMT status
Unmethyl.
Methyl.
B
Discovery cohort
p = 2.0x10−5
Validation cohort
p = 0.048
Radiosensitive signature ssGSEA score
Cohort
Discovery
Validation
Sample type
Primary
GBO
rGBO
Sensitive
Middle
Resistant
Sensitive
Middle
Resistant
C
UP-10311 Synergy score
Radiation (Gy)
GSK778 (μM)
Synergy score
D
UP-8017 Synergy score
Radiation (Gy)
GSK778 (μM)
Synergy score
E
UP-10311 Synergy score
Radiation (Gy)
ABBV-744 (μM)
Synergy score
F
UP-9057 Synergy score
Radiation (Gy)
ABBV-744 (μM)
Synergy score
G
UP-10311
UP-8017
UP-9057
Day 0
Day 8
DMSO
0.1 μM JQ-1
1 μM JQ-1
Control
8 Gy
H
UP-10311 Synergy score
Radiation (Gy)
JQ-1 (μM)
Synergy score
I
UP-8017 Synergy score
Radiation (Gy)
JQ-1 (μM)
Synergy score
J
UP-9057 Synergy score
Radiation (Gy)
JQ-1 (μM)
Synergy score

Figure S3. Identification of compounds to enhance radiotherapy efficacy in rGBOs, related to Figure 2 and Figure 3.
(A) Dot plots showing the correlation between the expression of the identified radiosensitive signature and *z*-scored AUC values of radiotherapy response, grouped by sample type and cohort.
(B) Boxplots showing the expression of the identified radiosensitive signature, stratified by rGBO radiotherapy response, similar to **Figure 2G**. Data points are colored by cohort, and point shapes indicate sample type. To account for samples from the same patient but different types, we computed *p*-values via a linear mixed-effects model fit by maximum likelihood, with patient number as a random variable.
(C-F) 3D surface plots showing the ZIP synergy scores of GSK778 and ABBV-744 treatment with radiation in rGBOs.
(G) Sample bright-field images of GBOs with JQ-1 treatment and radiotherapy. Scale bars, 100 µm. **(H-J)** 3D surface plots showing the ZIP synergy scores of JQ-1 treatment with radiation in GBOs from 10311 (**H**), 8017 (**I**) and 9057 (**J**). Synergy scores are calculated using GBO sizes and normalized to control conditions.

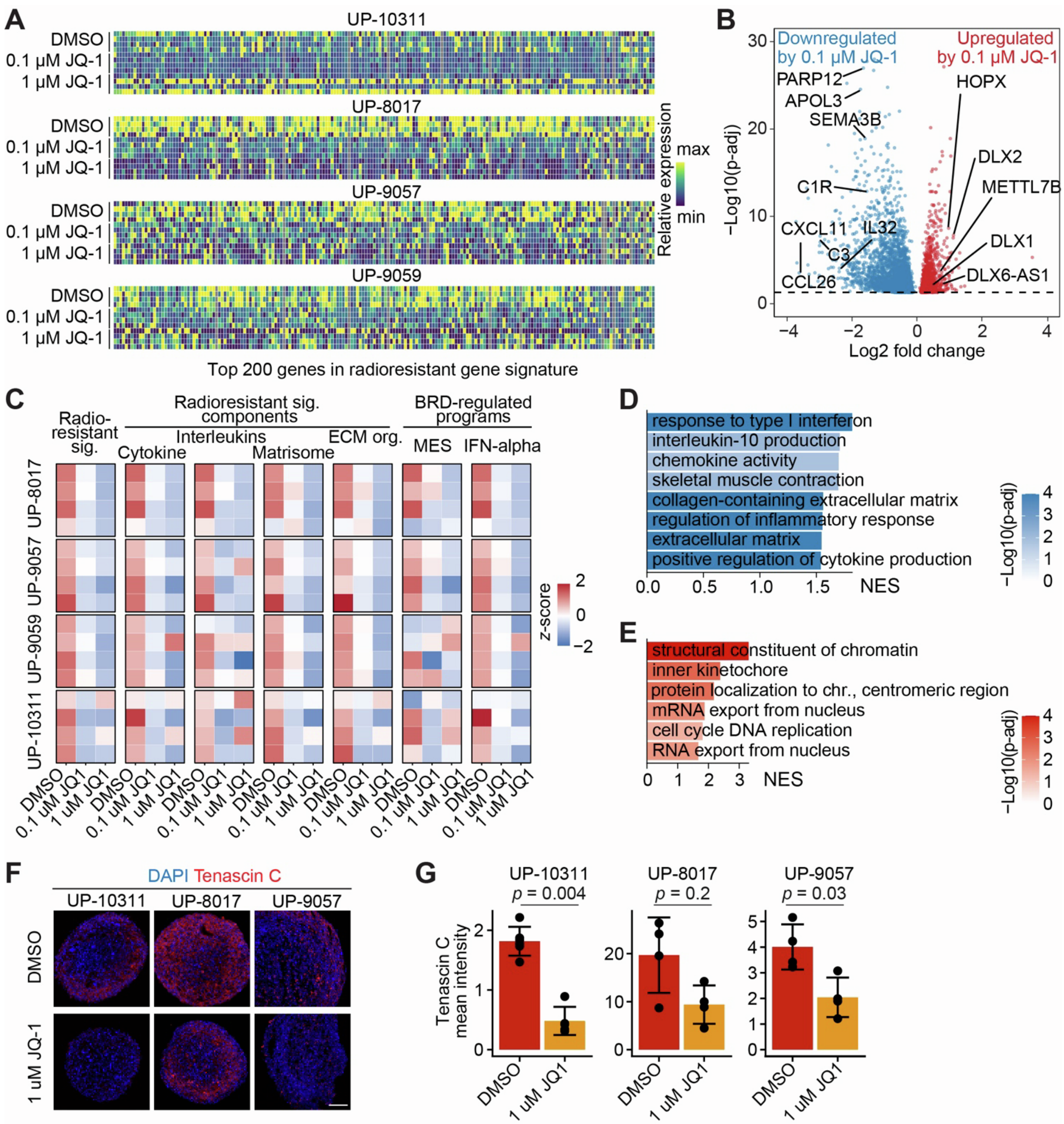
A
UP-10311
DMSO
0.1 µM JQ-1
1 µM JQ-1
UP-8017
UP-9057
UP-9059
Relative expression
max
min
Top 200 genes in radioresistant gene signature
B
Downregulated by 0.1 µM JQ-1
Upregulated by 0.1 µM JQ-1
PARP12
APOL3
SEMA3B
C1R
CXCL11
C3
IL32
CCL26
HOPX
DLX2
METTL7B
DLX1
DLX6-AS1
−Log10(p-adj)
Log2 fold change
C
Radio-resistant sig.
Radioresistant sig. components
Cytokine
Interleukins
Matrisome
ECM org.
BRD-regulated programs
MES
IFN-alpha
z-score
DMSO
0.1 uM JQ1
1 uM JQ1
D
response to type I interferon
interleukin-10 production
chemokine activity
skeletal muscle contraction
collagen-containing extracellular matrix
regulation of inflammatory response
extracellular matrix
positive regulation of cytokine production
NES
−Log10(p-adj)
E
structural constituent of chromatin
inner kinetochore
protein localization to chr., centromeric region
mRNA export from nucleus
cell cycle DNA replication
RNA export from nucleus
F
DAPI Tenascin C
UP-10311
UP-8017
UP-9057
DMSO
1 uM JQ-1
G
UP-10311
p = 0.004
UP-8017
p = 0.2
UP-9057
p = 0.03
Tenascin C mean intensity
DMSO
1 uM JQ1

Figure S4. Mechanistic investigation of the synergistic effect of JQ-1 on radiotherapy, related to Figure 4.
(A) Heatmaps showing the expression of the top 200 radioresistant DEGs in rGBOs from n = 4 patients (represented in each row), treated with different conditions (DMSO, 0.1 μM JQ-1, and 1 μM JQ-1; three replicates per condition).
(B) Volcano plot showing upregulated (red) or downregulated (blue) DEGs following 0.1 μM JQ-1 treatment compared with control condition (GBOs from n = 4 patients).
(C) Heatmaps showing the expression of the radioresistant signature, radioresistant signature components, and published BRD-regulated transcriptional programs[51,52] in rGBOs from n = 4 patients (represented in each row), treated with different conditions (DMSO, 0.1 μM JQ-1, and 1 μM JQ-1; n = 4 replicates per condition). Radioresistant signature components are obtained by selecting genes overlapping with known gene set pathways in Metascape.[44] sig., signature; org., organization; MES, mesenchymal.
(D-E) GO terms enriched for downregulated (**D**) and upregulated DEGs (**E**) following 0.1 μM JQ-1 treatment compared with the control condition. NES, normalized enrichment score.
(F-G) Sample images (**F**) and quantification (**G**) of Tenascin C in GBOs (n = 3 patients with $\geqslant$ 4 replicates per condition). Statistical significance between conditions is assessed using two-tailed Wilcoxon tests. Scale bars, 100 μm.
